# Enhancing random forest predictive performance for foot and mouth disease outbreaks in Uganda: a calibrated uncertainty prediction approach for varying distributions

**DOI:** 10.3389/frai.2024.1455331

**Published:** 2024-11-01

**Authors:** Geofrey Kapalaga, Florence N. Kivunike, Susan Kerfua, Daudi Jjingo, Savino Biryomumaisho, Justus Rutaisire, Paul Ssajjakambwe, Swidiq Mugerwa, Seguya Abbey, Mulindwa H. Aaron, Yusuf Kiwala

**Affiliations:** ^1^Department of Information Technology, College of Computing and Information Sciences, Makerere University, Kampala, Uganda; ^2^Department of Vaccinology, National Livestock Resources Research Institute, Kampala, Uganda; ^3^African Center of Excellence in Bioinformatics (ACE-B), Makerere University, Kampala, Uganda; ^4^Department of Computer Science, College of Computing and Information sciences, Makerere University, Kampala, Uganda; ^5^College of Veterinary Medicine, Animal Resources and Bio-security, Makerere University, Kampala, Uganda; ^6^College of Business and Management Science, Makerere University, Kampala, Uganda

**Keywords:** foot-and-mouth disease, random forest, distribution shifts, performance improvement rates, calibrated uncertainty prediction

## Abstract

Foot-and-mouth disease poses a significant threat to both domestic and wild cloven-hoofed animals, leading to severe economic losses and jeopardizing food security. While machine learning models have become essential for predicting foot-and-mouth disease outbreaks, their effectiveness is often compromised by distribution shifts between training and target datasets, especially in non-stationary environments. Despite the critical impact of these shifts, their implications in foot-and-mouth disease outbreak prediction have been largely overlooked. This study introduces the Calibrated Uncertainty Prediction approach, designed to enhance the performance of Random Forest models in predicting foot-and-mouth disease outbreaks across varying distributions. The Calibrated Uncertainty Prediction approach effectively addresses distribution shifts by calibrating uncertain instances for pseudo-label annotation, allowing the active learner to generalize more effectively to the target domain. By utilizing a probabilistic calibration model, Calibrated Uncertainty Prediction pseudo-annotates the most informative instances, refining the active learner iteratively and minimizing the need for human annotation and outperforming existing methods known to mitigate distribution shifts. This reduces costs, saves time, and lessens the dependence on domain experts while achieving outstanding predictive performance. The results demonstrate that Calibrated Uncertainty Prediction significantly enhances predictive performance in non-stationary environments, achieving an accuracy of 98.5%, Area Under the Curve of 0.842, recall of 0.743, precision of 0.855, and an F1 score of 0.791. These findings underscore Calibrated Uncertainty Prediction’s ability to overcome the vulnerabilities of existing ML models, offering a robust solution for foot-and-mouth disease outbreak prediction and contributing to the broader field of predictive modeling in infectious disease management.

## 1 Introduction

*Foot-and-mouth disease* (FMD) remains a formidable challenge which directly and indirectly affects the livestock industry, communities and the economy (Kerfua, 2020; Munsey et al., 2019). The disease circulates in approximately 77% of the global livestock population, primarily in Africa, the Middle East, and Asia (Bachanek-Bankowska et al., 2018; Zewdie et al., 2023), causing significant annual economic losses estimated between US$6.5 to 21 billion (Knight-Jones and Rushton, 2013). In Uganda, the disease has persisted for over six decades leading to 83 and 88% reductions in market values for bulls and cows, respectively, during FMD outbreaks (Baluka, 2016). The author further acknowledged a 23% decline in income for livestock industry stakeholders at the processing plants. The country embraces a reactive approach in managing FMD outbreaks where current interventions including vaccination, restriction on livestock movement and quarantine measures are implemented (Munsey et al., 2019). Due to the contagious nature of the disease, such interventions have had limited impact on control efforts, partly because the disease is often detected too late, after it has already spread to other regions of the country (Kerfua et al., 2013; Mwiine et al., 2019).

**Figure 1:**
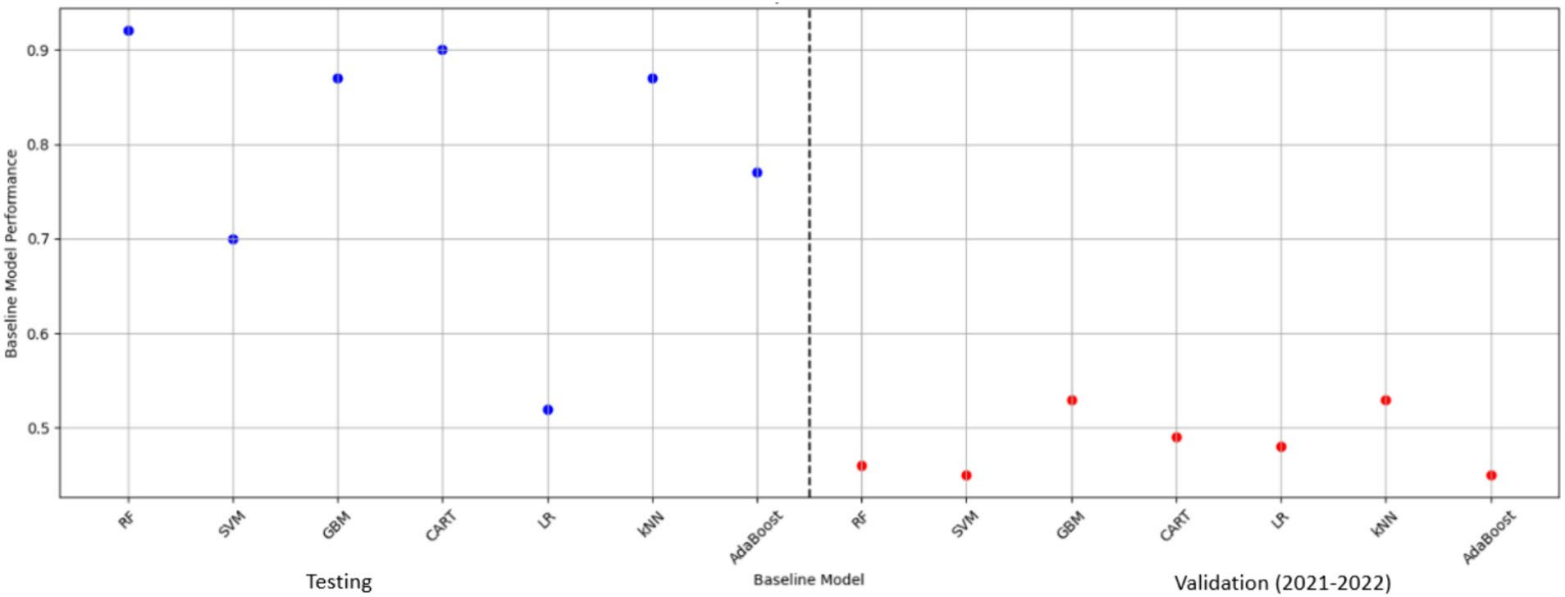
Model accuracy degradation under varying distributions. RF, random forest; SVM, support vector machine; kNN, k-nearest neighbors; GBM, gradient boosting machine; AdaBoost, adaptive boost; LR, logistic regression; CART, classification and regression tree.

**Figure 2:**
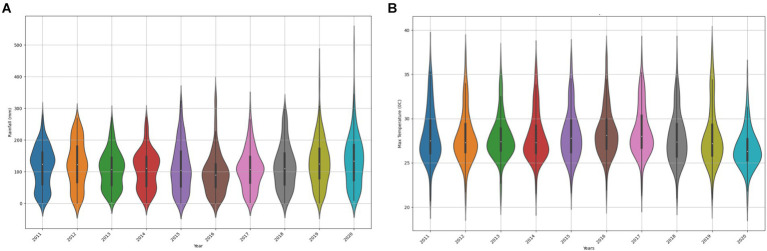
Variability in rainfall **(A)** and max temperature **(B)** features, highlighting varying distribution (Kapalaga et al., 2024).

Random Forest (RF), an ensemble machine learning (ML) algorithm, has been used to predict FMD outbreaks in stationary environments, where the distribution of the training and test datasets is similar. This approach enables early detection of the virus and optimal allocation of resources (Punyapornwithaya et al., 2022). In such stationary settings, RF has exhibited high predictive performance due to its ensemble nature, where multiple decision trees are constructed and combined to make robust predictions using bagging (Mosavi et al., 2021). Each tree is trained on a random subset of the data and features, which helps to reduce overfitting and increase generalizability. However, in non-stationary environments with varying distributions (distribution shifts), RF demonstrates significant degradation in performance as depicted in Figure 1 (Kapalaga et al., 2024), rendering it unsuitable for deployment in the endemic and dynamic nature of Uganda. Its poor performance was attributed to the high variability in rainfall and maximum temperatures (Figure 2), which are key factors influencing FMD outbreaks. While several methods exist to mitigate distribution shifts, they often fail when the shifts are significant (Gulrajani and Lopez-Paz, 2020; Koh et al., 2021). Techniques such as pre-training and data augmentation can generate inconsistent results across different datasets (Wiles et al., 2021). Moreover, these methods require labeled datasets, which are time-consuming and costly to acquire (Kouw and Loog, 2019), and sometimes expert human annotators are unavailable (Settles, 2009; Yang and Loog, 2019).

In this study, two primary objectives were proposed: (1) to develop a Calibrated Uncertainty Prediction (CUP) approach for enhancing RF model performance under varying distributions, and (2) to evaluate the performance of CUP in mitigating distribution shifts for predicting FMD outbreaks in the dynamic setting of Uganda. The study contributes to both practical applications and methodological advancements. Practically, the proposed approach enables proactive measures by providing timely and accurate predictions, facilitating early detection of outbreaks, and optimal resource allocation for managing FMD, thereby safeguarding livestock, the economy, and the community. Methodologically, the CUP approach addresses distribution shifts challenge, which is reported to degrade performance for RF-based prediction of FMD outbreaks (Kapalaga et al., 2024).

The rest of the paper is structured as follows: Section 2 offers an in-depth review of the relevant literature, highlighting key studies and identifying the research gap. Section 3 outlines the materials and methods used in the study, detailing the experimental design, data collection, and analytical techniques employed. Section 4 presents the results, providing a thorough analysis of the data and key findings. In Section 5, the findings are discussed, interpreting the results in the context of existing research and the study’s objectives. Finally, Section 6 concludes the paper, summarizing the key insights and implications of the research, and suggesting directions for future work.

## 2 Literature review

In this section, the study briefly defines DS, their causes, and current methods attempting to address them, along with their limitations. Additionally, the study highlights recent developments in related research areas that, when integrated, can effectively address distribution shifts in ML-based prediction of FMD outbreaks in the dynamic setting of Uganda.

### 2.1 Definition of DS and causes

DS, also called dataset shift or domain shift is a common problem in ML-based predictive modeling that occurs when training and test joint distributions are different (Ovadia et al., 2019). The concept of DS was initially introduced in the book by (Quinonero-Candela et al., 2008), which marked the first comprehensive compilation in this field. In this seminal work, DS was defined as instances where the joint distribution of inputs and outputs varies between the training and testing stages (Storkey, 2009). DS can arise from various factors, namely sample selection bias and non-stationary environments (Castle et al., 2021; Moreno-Torres et al., 2012). Sample selection bias occurs when training examples are obtained through biased methods, leading to a discrepancy in distribution and a lack of representation of the operational environment where the classifier will be deployed (Liu et al., 2021). Non-stationary environments, arises when the training environment differs from the test environment due to temporal or spatial changes (Sugiyama and Kawanabe, 2012).

Mathematically, DS is the alterations in the joint distribution of P(X, Y), where X denotes the predictors and Y represents the targets of a ML model (Quinonero-Candela et al., 2008). Such alterations in P(X, Y) can stem from changes in P(X), P(Y), or P(Y|X). These distinct alterations are often referred to using varied terminology by different authors. However, Jose G. Moreno-Torres proposed a more unified naming convention, labeling changes to P(X) as covariate shift, changes to P(Y) as prior probability shift, and changes to P(Y|X) as concept shift (Moreno-Torres et al., 2012).

### 2.2 Predictive performance degradation for FMD outbreaks under varying distribution

In our previous study, we quantified the influence of distribution shifts on the predictive performance of ML-based algorithms for FMD outbreaks (Kapalaga et al., 2024). RF which had demonstrated superb predictive performance under similar distribution, experienced a significant decrease across all performance metrics. Its accuracy decreased by 50% and a notable decline of 40.21% in the Area Under the Curve (AUC) value of the Receiver Operating Characteristic (ROC) curve. Similarly, RF experienced reductions in Recall by 96.81%, Precision by 73.33%, and F1-score by 93.48%. These performance degradation rates in prediction of FMD under varying distributions underscore the need for high performing methods to address the challenge.

### 2.3 Methods for addressing DS and their limitations

**Figure 3:**
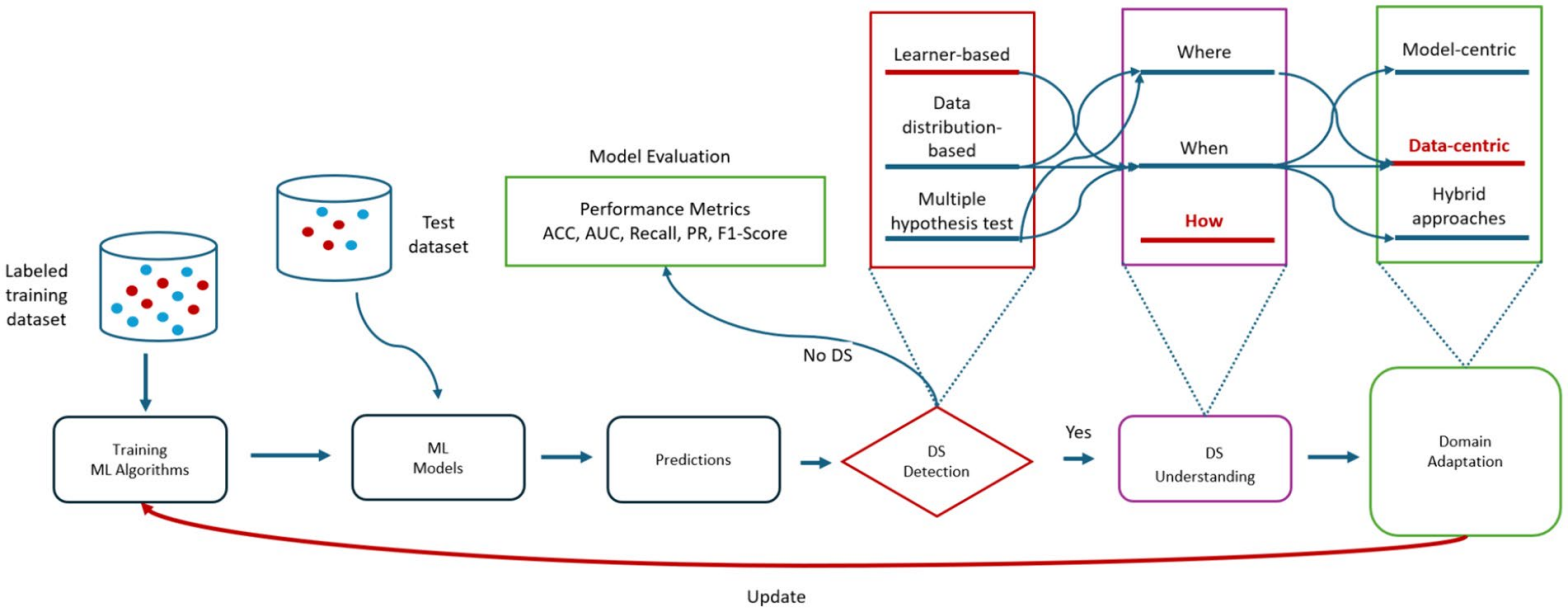
A general framework for handling distribution shifts in ML. ACC, accuracy; AUC, area under curve; PR, precision; DS, distribution shifts; ML, machine learning.

DA is a specialized technique in ML designed to address the challenge of distribution shift (DS), which occurs when the data distribution in the target domain differs from that in the source domain. This shift can significantly impair the performance of ML models, making it a critical issue to address. The general framework for dealing with DS in ML, as illustrated in Figure 3, encompasses various strategies aimed at adapting models to new data distributions.

One of the most common approaches to mitigating DS involves acquiring unlabeled data from the target domain and utilizing it to fine-tune models. This method is particularly beneficial when labeled data in the target domain is scarce or unavailable. DA techniques can be classified according to the availability of labels in the target domain. In supervised domain adaptation, labeled data from the target domain is available, allowing models to learn directly from the target distribution (Motiian et al., 2017). In semi-supervised domain adaptation, a small amount of labeled data from the target domain is available alongside a larger pool of unlabeled data, which helps refine model performance (Berthelot et al., 2021). Unsupervised domain adaptation takes on the challenge of adapting models when only unlabeled data from the target domain is available, making it a particularly challenging area of research (Kang et al., 2019). A more recent approach, pseudo-semi-supervised domain adaptation, involves generating pseudo-labels for the target domain’s unlabeled data, which are then used to guide the adaptation process (Singhal et al., 2023)(Singhal et al., 2023).

Addressing DS can also be approached differently depending on the focus of adaptation. Model-centric approaches concentrate on modifying the model architecture or learning algorithm to accommodate shifts in data distribution. This might involve adding domain-specific layers or parameters to the model, which are designed to better handle the differences between source and target domains (Dou et al., 2019; Ramponi and Plank, 2020). On the other hand, data-centric approaches involve transforming the data itself, such as through normalization, augmentation, or selecting specific features that are less sensitive to changes in data distribution. These transformations aim to make the data more consistent across different domains, thereby improving model performance (Bashath et al., 2022; Liang et al., 2019). Hybrid approaches combine both model-centric and data-centric techniques, leveraging the strengths of each to maximize the effectiveness of adaptation strategies (Amrani, 2021).

Several techniques are commonly employed to address DS. Maximum Mean Discrepancy (MMD) is a statistical method used to measure the difference between distributions and reduce this gap during model training (Long et al., 2016). Huber Loss is a loss function that is less sensitive to outliers, helping models adapt more effectively to changes in data distribution (Huber, 1992; Owen, 2007; Zwald and Lambert-Lacroix, 2012). Singular Value Decomposition (SVD) is a technique for dimensionality reduction that can help identify key features across different domains, thereby facilitating better model adaptation (Rebentrost et al., 2018). Latent Discriminant Analysis (LDA) is another method used for feature extraction and dimensionality reduction, making it easier for models to learn from data with varying distributions (Leng et al., 2020). Partial Least Squares (PLS) finds the fundamental relations between two matrices, aiding in domain adaptation (Gong et al., 2019). Additionally, KL Divergence is a metric used to rank domains based on their similarity, which can guide the adaptation efforts by identifying the most relevant domains for model training (Farahani et al., 2021). Lastly, Dynamic Weighted Majority (DWM) is an ensemble learning technique that adjusts the weights of base classifiers to better cope with DS, thereby improving the overall model performance in dynamic environments (Kolter and Maloof, 2007).

Despite these advancements, existing methods for addressing DS have notable limitations. A significant challenge is the substantial disparity between the source and target domains, which can result in poor model performance even after adaptation efforts (Gulrajani and Lopez-Paz, 2020; Koh et al., 2021). The scarcity of labeled target data exacerbates this issue, as acquiring such data often requires considerable time and financial resources. Moreover, the unavailability of expert annotators further complicates the situation, limiting the scope of effective domain adaptation (Settles, 2009; Yang and Loog, 2019). Even techniques like pretraining on large datasets and data augmentation, which are intended to mitigate DS, exhibit inconsistencies across different datasets. These inconsistencies suggest that there is no universal solution, and the effectiveness of these techniques varies depending on the specific characteristics of the datasets involved (Wiles et al., 2021). The challenges presented by DS underscore the need for novel approaches that can more effectively address this issue and enhance predictive performance in non-stationary environments. Developing strategies that integrate active learning, data augmentation, probabilistic calibration, and pseudo-labeling could provide more robust solutions for DS in dynamic settings, such as in the prediction of FMD outbreaks in Uganda. By improving the adaptation of ML models to dynamic distribution changes, this study aims to contribute to more accurate and effective disease control and management strategies.

### 2.4 Related research areas

#### 2.4.1 Data augmentation

ML models demand substantial data for effective learning and accurate predictions (Makridakis et al., 2018; Verbraeken et al., 2020). However, gathering and annotating large volumes of data is laborious and costly, posing a challenge in training models for real-world applications (Paleyes et al., 2022; Wu et al., 2022). Various data augmentation methods aim to diversify limited datasets, creating a more comprehensive representation of the target distribution. Augmentation techniques play a pivotal role in expanding and diversifying limited training data, ultimately improving a model’s generalization capacity and enriching its insights into the problem domain (Wang et al., 2023). The oversampling method has emerged as a crucial approach for augmenting data within a dataset, particularly focusing on boosting instances in the minority class. A variety of oversampling techniques are available, including the Synthetic Minority Over-sampling TEchnique (SMOTE; Original; Chawla et al., 2002), Borderline-SMOTE (Han et al., 2005), Adaptive Synthetic Sampling (ADASYN; He et al., 2008), SMOTE with Edited Nearest Neighbors (SMOTE-ENN; Muntasir Nishat et al., 2022), Safe-Level SMOTE (Bunkhumpornpat et al., 2009), Borderline-SMOTE SVM (Synthetic Minority Oversampling Technique-Support Vector Machine; Wang et al., 2017), K-Means SMOTE (Douzas et al., 2018) and Random Oversampling (Mohammed et al., 2020), among others. Research indicates the potential of oversampling to improve predictive model performance in various domains of ML application (Barfungpa et al., 2024; Karamti et al., 2023; Priyadarshinee and Panda, 2022). In our previous study, Borderline-SMOTE was the best performing oversampling technique in mitigating the class imbalance exhibited during prediction of FMD in a stationary environment (Kapalaga et al., 2024). However, oversampling methods may introduce biases and lead to overfitting (Huda et al., 2018; Koziarski et al., 2019; Vandewiele et al., 2021). Data augmentation effectiveness can also diminish if the disparities between source and target domains are substantial (Antoniou et al., 2017; Shorten and Khoshgoftaar, 2019).

#### 2.4.2 Active learning (AL)

AL is a subfield of ML aimed at reducing annotation costs and improving learning performance by iteratively selecting the most informative samples for labeling (Budd et al., 2021; Ren et al., 2021). Despite the necessity for large labeled datasets in ML, acquiring labels is time-consuming and costly (Ren et al., 2021), especially in real-world applications like disease outbreak annotation (Polonsky et al., 2019). AL addresses this challenge by selecting informative samples for labeling, thus reducing annotation costs while maintaining learning performance (Monarch and Munro, 2021). Pool-based active learning is prevalent across various domains, where extensive collections of unlabeled data are simultaneously available (Bhatnagar et al., 2020; Chandrasekaran et al., 2020; Karlos et al., 2021; Lowell et al., 2018; Schröder and Niekler, 2020; Zhan et al., 2021). In this approach, a small set of labeled data is augmented iteratively by selecting informative instances from a pool of unlabeled data. Uncertainty sampling is a widely used query framework in active learning, selecting instances based on the model’s uncertainty in labeling (Nguyen et al., 2022; Ren et al., 2021).

In this study, we adopt uncertainty sampling as the sample selection strategy for active learning due to its simplicity, effectiveness, and flexibility across different probabilistic models (Bull et al., 2019; Kottke et al., 2021; Settles, 2011). This approach aligns with our goal of enhancing the predictive performance of FMD model in dynamic environments where distribution shifts is prevalent in key predictor like rainfall and maximum temperature. By prioritizing instances where the model’s confidence is low, uncertainty sampling optimizes the efficiency of the active learning process and improves the FMD model’s performance.

#### 2.4.3 Pseudo-label annotation (PLA)

PLA diverges from AL by leveraging a pre-trained model on labeled source data to predict labels for unlabeled target data in batches (Rizve et al., 2021; Shin et al., 2020). Unlike AL, where human annotation is involved, pseudo-labeling methods rely solely on model predictions (Arazo et al., 2020; Cascante-Bonilla et al., 2021; Ding et al., 2018). Although the labels assigned to the target data are not entirely accurate (Arazo et al., 2020; Wang et al., 2022), they mirror the labeled source data to some extent (Cho et al., 2022; Pham et al., 2021; Zou et al., 2020). One common approach is to incorporate these pseudo-labeled target samples alongside the labeled source data to train a new model (Liang et al., 2019; Shin et al., 2020; Wang and Breckon, 2020). However, this method is susceptible to the introduction of noisy or incorrect labels, which can adversely affect model performance (Park et al., 2020; Rizve et al., 2021; Wang et al., 2022). In addressing the challenge of noisy labels, various techniques have been proposed in different domains (Huang et al., 2021; Liang et al., 2021; Wang and Breckon, 2020). Despite these efforts to address noisy label problems, there remains inconsistency in performance (Arazo et al., 2020; Cascante-Bonilla et al., 2021, 2021). This underscores the necessity for further exploration and experimentation in this area.

#### 2.4.4 Probabilistic calibration (PC)

PC aims to convert prediction scores from ML models into reliable probability estimates (Hébert-Johnson et al., 2018; Vaicenavicius et al., 2019). Various techniques exist, including empirical binning calibration, isotonic regression, Platt scaling, probability calibration trees, beta calibration, and temperature scaling (Kull et al., 2017; Wenger et al., 2020). These methods adjust prediction scores to ensure they represent accurate probabilities, improving model interpretability and performance. Despite advancements in calibration techniques, current models often struggle with generalization to distribution shifts (Gulrajani and Lopez-Paz, 2020; Koh et al., 2021). The dynamic nature of deployment environments, such as those encountered in FMD dataset, presents challenges in handling distribution shifts (Settles, 2009). The limited generalization to distribution shifts can lead to false alarms and the need for costly and time-consuming labeling efforts by domain experts. Therefore, there is a pressing need for more advanced distribution shift applications to address these challenges and improve model robustness in dynamic environments like FMD prediction.

DA addresses the challenge of domain shift in ML by adapting a model trained on a source domain to perform better in a target domain (Jing et al., 2020; Sun et al., 2016; Wilson and Cook, 2020). Methods for DA vary based on the availability of labels in the target domain: supervised DA, semi-supervised DA, unsupervised DA, and pseudo-semi-supervised DA (Motiian et al., 2017; Singhal et al., 2023). DA methods are mainly categorized into model-centric, data-centric and hybrid approaches as illustrated in Figure 3.

Data-centric DA strategies leverage intrinsic data characteristics rather than modifying model architecture or loss functions (Fan et al., 2022). Techniques like pseudo-labeling automatically assign labels to unlabeled data using pre-trained models, treating inferred labels as training data (Singhal et al., 2023). Data selection methods aim to identify source domain data closely aligned with the target domain, but this area remains underexplored despite past applications in machine translation. Pre-training, particularly with large Transformer-based models, is a prevalent method in Natural Language Processing (NLP) domain adaptation (Kalyan et al., 2021), but challenges persist, including inconsistent model performance across datasets and limited improvement under varying distributions (Gulrajani and Lopez-Paz, 2020; Heaven, 2020; Koh et al., 2021; Wiles et al., 2021).

### 2.5 Research gap

The current ML-based research on FMD prediction largely focuses on stationary environments, neglecting the critical challenge of distribution shifts in non-stationary settings. This oversight leaves predictions vulnerable to unexpected changes, reducing their reliability. While domain adaptation techniques, particularly in computer vision, have made progress, they struggle with large disparities between source and target domains, especially when labeled target data is scarce and expensive to acquire. Moreover, methods like pre-training and data augmentation show inconsistent results across different datasets and distribution shifts. This gap underscores the need for innovative approaches that address distribution shifts and improve prediction performance in non-stationary environments. To fill this gap, this study explores a CUP approach. The CUP integrates borderline-SMOTE, active learning, probabilistic calibration, and pseudo-labeling to effectively manage varying distributions in a curated FMD dataset. This approach aims to enhance the robustness and accuracy of predictions under dynamic conditions in Uganda, contributing to better disease control and resource allocation for FMD management.

## 3 Materials and methods

### 3.1 Employing an experimental design to conduct the study

**Figure 4:**
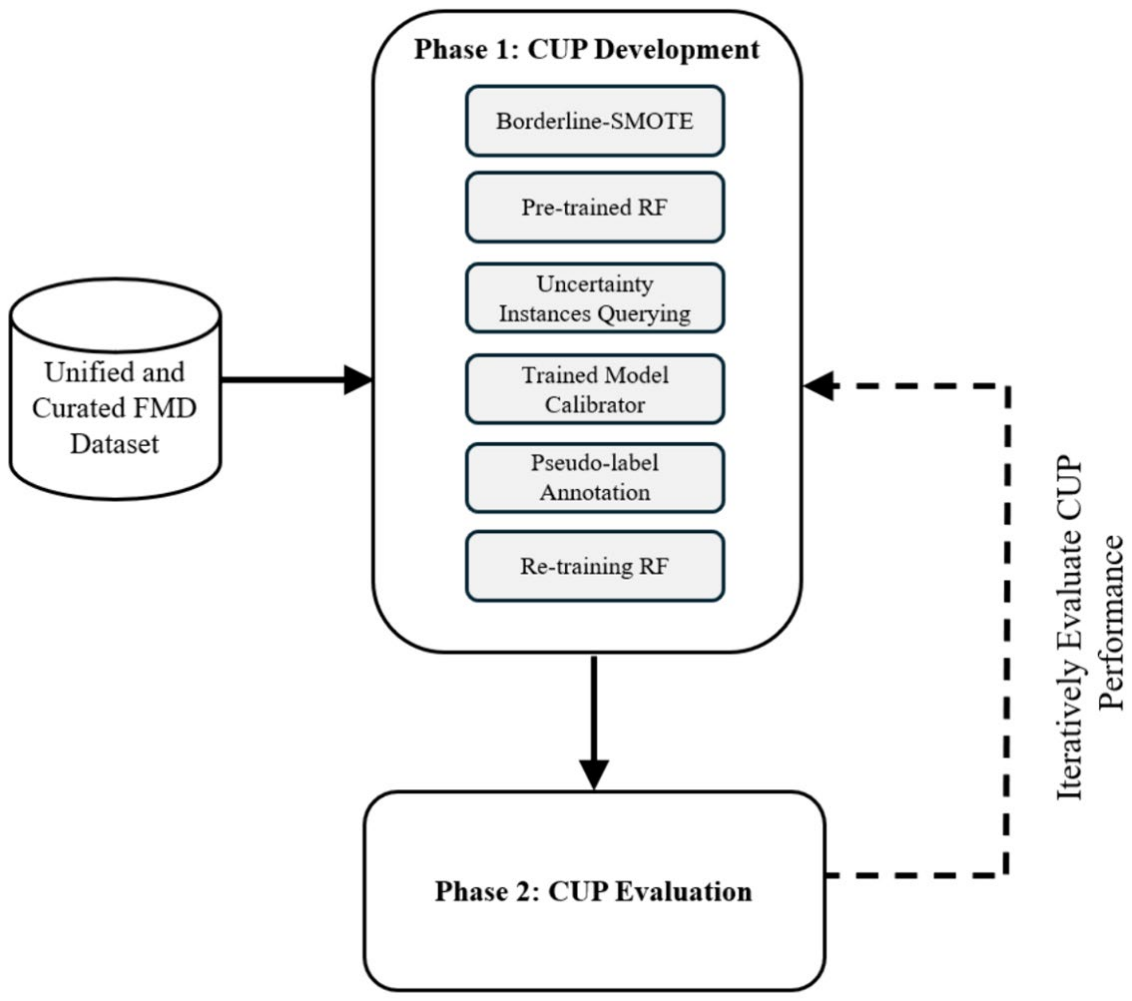
Experimental design to guide the CUP development and evaluation. RF, random forest; CUP, calibrated uncertainty prediction; FMD, foot-and-mouth disease.

To achieve the main goal of enhancing predictive performance of RF model for FMD outbreaks under varying distributions, the study employed an experimental research design to develop and evaluate a CUP approach. Experimental research design in ML involves a structured approach for planning, executing, and analyzing experiments (Kamiri and Mariga, 2021). The methodology as depicted in Figure 4, ensured a rigorous design, development and evaluation of the proposed CUP method in enhancing predictive performance rates in dynamic setting for FMD outbreaks. By employing various performance metrics including accuracy (ACC), AUC of the Receiver Operating Characteristic (ROC), recall, precision and F1-score, the study provides a comprehensive performance evaluation of the CUP approach’s effectiveness in addressing the challenges posed by distribution shifts in the unified and curated FMD dataset. The phases in the methodological approach include CUP development and CUP evaluation. Table 1 summarizes the key activities, methods, and descriptions used to achieve the study objectives.

**Table 1:** A Summary of the research phases, activities, methods and description of the methods for achieving the objectives.

Phase No.	Activity	Study objective	Research methods	Description
1	Develop a CUP approach to enhance RF model predictive performance for FMD outbreaks under varying distributions	Objective 1	Experiments	A CUP approach based on a data-centric domain adaptation involved integrating active learning, borderline-SMOTE, probabilistic calibration, and pseudo-label annotation.
2	Evaluate predictive performance of the proposed CUP approach in prediction of FMD outbreaks under varying distributions	Objective 2	Experiments	Five performance metrics including ACC, AUC, recall, precision, and F1-score were used to assess the performance of the proposed approach in comparison with existing methods reported to address distribution shifts in the ML domain.

#### 3.1.1 Data collection

**Figure 5:**
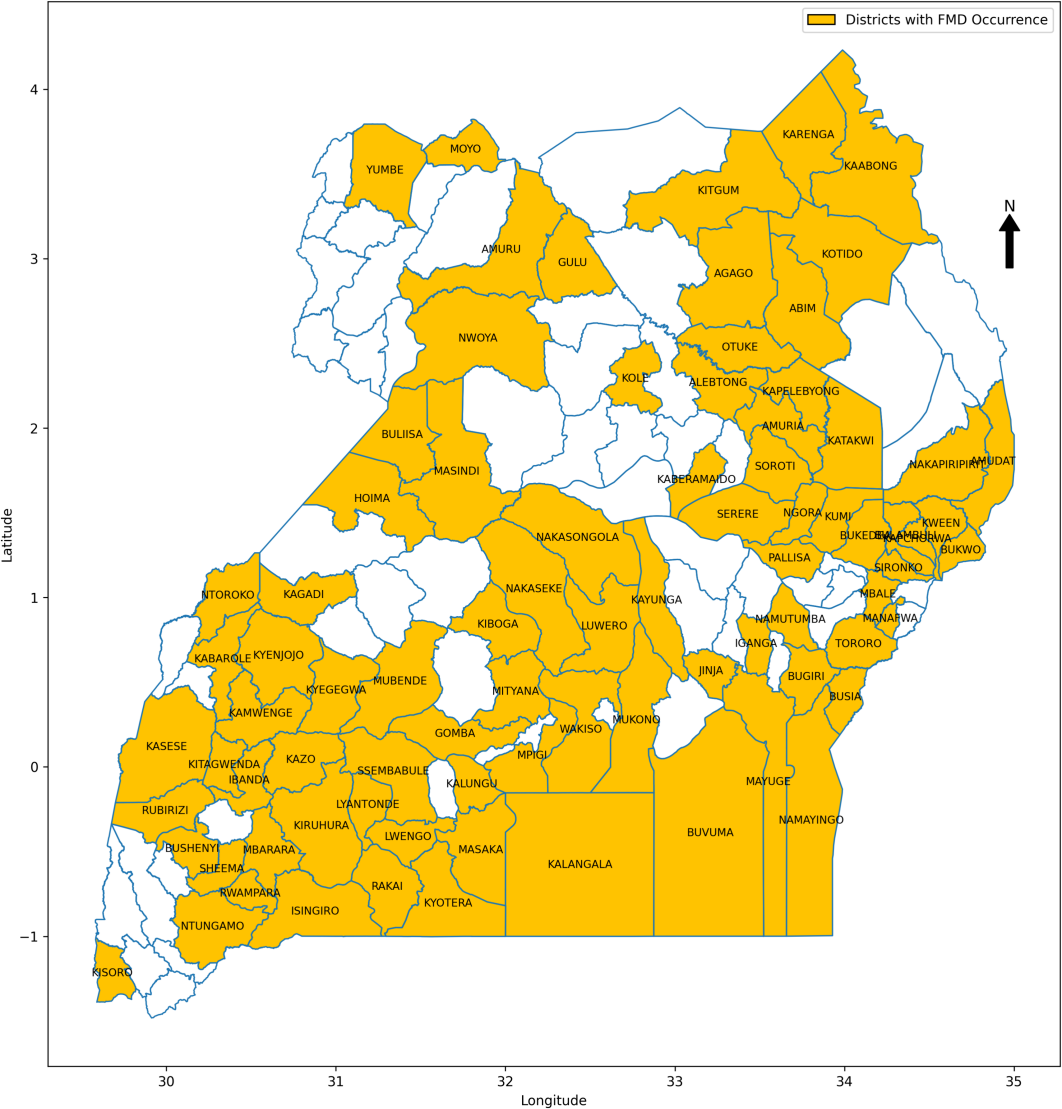
Map of Uganda showing districts affected by FMD outbreaks between 2011 and 2022.

This study focused on Uganda, an East African country with diverse landscapes and climates that contribute to varied ecological conditions affecting the transmission dynamics of FMD (Mwiine et al., 2019). The country’s geography, spanning savannahs, forests, and mountains, coupled with its tropical climate, plays a significant role in influencing the occurrence and spread of FMD outbreaks. A retrospective approach was employed to collect data from 2011 to 2022, drawing from multiple sources to create a comprehensive dataset for training and validating ML models for FMD prediction. The dataset included FMD outbreak records from 86 districts (Figure 5), obtained from the National Animal Disease Diagnostics and Epidemiology Centre (NADDEC) and the World Organisation for Animal Health (WOAH). The data captured essential details such as outbreak locations, timing, and confirmed cases. Additionally, the study incorporated climatic data including rainfall and temperature from the Uganda National Meteorological Authority (UNMA) and livestock population densities from the National Livestock Census 2008, conducted by the Ministry of Agriculture, Animal Industry, and Fisheries (MAAIF) and the Uganda Bureau of Statistics (UBOS). Geographical information related to proximity to protected wildlife zones and international borders was also included, as these factors significantly influence FMD transmission dynamics. The independent variables (risk factors) and their corresponding data sources are summarized in Table 2, with the presence of FMD outbreaks serving as the dependent variable.

**Table 2:** Composition of FMD dataset for the study.

Independent variables	Description	Source(s)	Data type	Minimum value	Maximum value	Dependent variable	Total records	Non-outbreaks	Outbreaks
Rainfall (mm)	Total amount of precipitation that falls in a district over the course of that month	UNMA	Continuous	0.0 mm	507.2 mm	Confirmed outbreak (1,0)	3,456	3,323 (96.15%)	133 (3.85%)
max_temp (degrees Celsius)	Maximum temperature recorded within a specific month	UNMA	Continuous	19.9°C	38.3°C				
cattle_density (cattle per square kilometer)	Number of cattle per square kilometer	UBOS & MAAIF	Continuous	13,635	674,746				
adjacent_national_parks (1,0)	District (s) that share a border with one or more national parks.	Pennsylvania State University	Categorical						
adjacent_international_border (1,0)	District(s) sharing borders with our countries.	Pennsylvania State University	Categorical						

##### 3.1.1.1 Data pre-processing

In this study, a comprehensive data pre-processing strategy was implemented to create an integrated FMD dataset for training, testing, and evaluating ML models for predicting FMD outbreaks in Uganda. This process involved several critical steps to ensure the accuracy, consistency, and reliability of the dataset.

Initially, missing values in the dataset were addressed using mean imputation, a technique where missing data points are replaced with the mean value of their respective features (Van Ginkel et al., 2020). This method was selected for its simplicity and effectiveness in maintaining dataset completeness, ensuring that essential variables were preserved for subsequent analysis and model development. Duplicate records, which can introduce bias and reduce the reliability of the analysis, were identified and removed using Python’s Pandas library (Pandas—Python Data Analysis Library, 2024). This process ensured that the dataset was free from redundancy, thereby enhancing its integrity and the accuracy of the models built upon it. Outliers, which could potentially skew the results, were detected using the Z-score method (Chikodili et al., 2020). Confirmed outliers were treated by replacing them with the mean value of the respective feature. This approach maintained the consistency and reliability of the dataset, ensuring that extreme values did not adversely affect the predictive modeling process. Data integration was another critical step, where multiple datasets from various sources, including historical FMD outbreak records and environmental data, were merged into a single, cohesive dataset. This was achieved using Python’s pd.merge function, which facilitated the seamless integration of data based on common identifiers. This integrated dataset (Table 2) provided a comprehensive foundation for in-depth analysis and modeling. Feature engineering played a vital role in enhancing the performance of the ML models. New features, including monthly rainfall and monthly maximum temperature, were created by summing daily rainfall values and selecting the maximum temperature, respectively, to align with the monthly FMD outbreak data. These engineered features were crucial in improving the predictive performance of the model. Finally, categorical data encoding was employed to convert qualitative variables including outbreak occurrences, into numerical formats suitable for ML algorithms. The target variable was encoded to represent outbreak (1) and non-outbreak (0) instances, facilitating the interpretation and modeling of FMD outbreaks. Through these data pre-processing steps, the study ensured that the integrated dataset was well-prepared for accurate and robust predictive modeling, ultimately contributing to the effective prediction and management of FMD outbreaks in Uganda.

##### 3.1.1.2 Descriptive analysis of pre-processed FMD dataset

**Figure 6:**
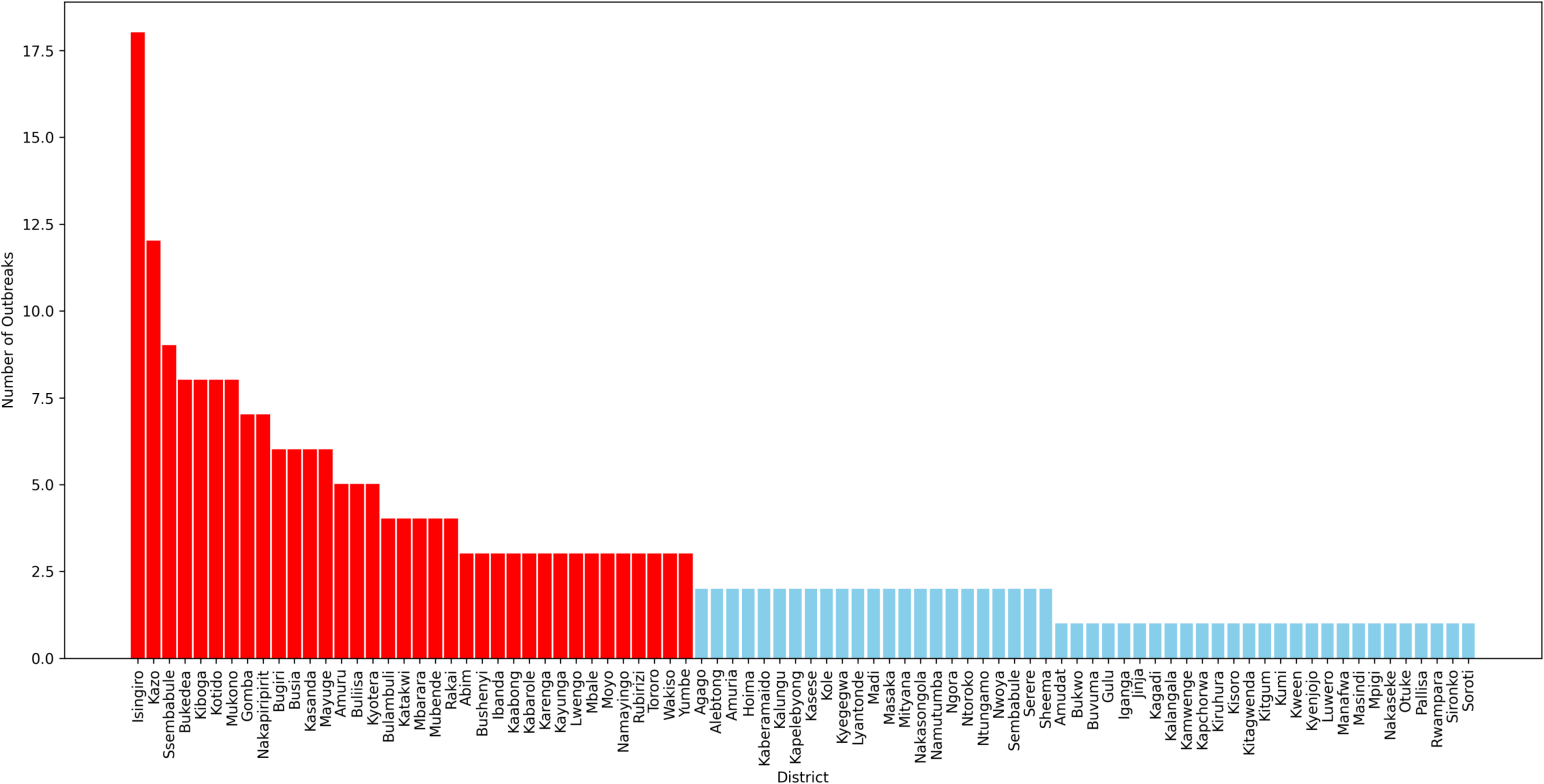
Prevalence of FMD outbreaks by district.

The pre-processed dataset for FMD in Uganda contains a total of 12,384 records, collected from 86 districts across the country. Each record represents either the occurrence or non-occurrence of an FMD outbreak in a given district within a specified time frame. The dataset reveals a significant class imbalance, with 97.88% of the records corresponding to non-outbreaks, and only 2.12% indicating outbreaks. This severe class imbalance is a critical factor to consider during the development and evaluation of ML models, as it can lead to biased predictions if not properly addressed. Further analysis of the dataset reveals considerable variation in the prevalence of FMD outbreaks across different districts, as depicted in Figure 6. Some districts report higher incidences of outbreaks, while others rarely experience them. This spatial disparity underscores the importance of incorporating geographical factors and local conditions into predictive models, as these variations can significantly influence the risk of outbreaks.

The descriptive statistics of the dataset provide a foundational understanding of the distribution and characteristics of the data. These insights are essential for guiding the selection of appropriate modeling techniques, particularly those that can effectively manage class imbalance and leverage the spatial heterogeneity observed in the data. By carefully considering these factors, the analysis aims to enhance the predictive performance and reliability of models used for forecasting FMD outbreaks, ultimately contributing to better disease management and control strategies in Uganda.

##### 3.1.1.3 Data sampling

**Figure 7:**
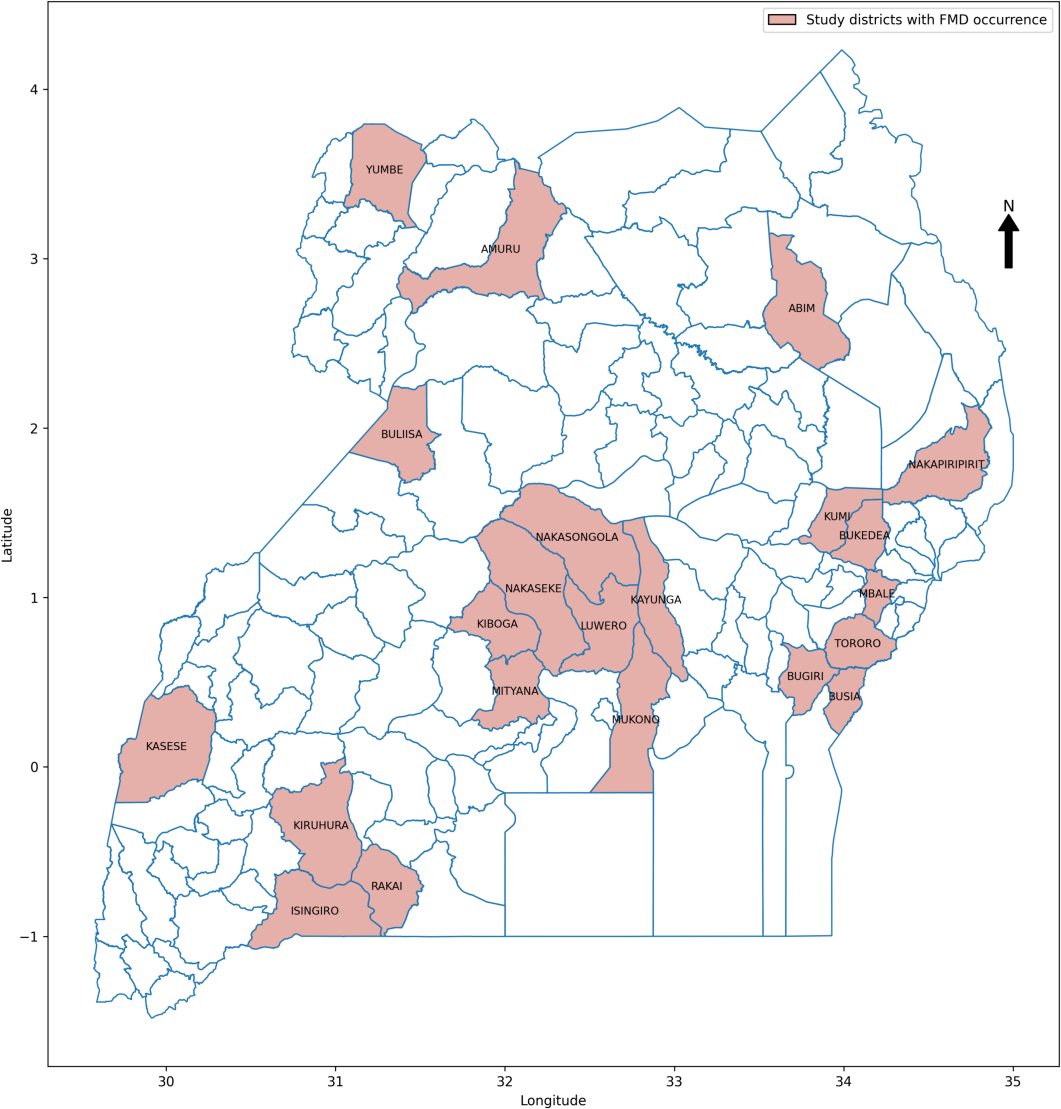
Map of Uganda with purposively selected study districts.

In data science, various sampling techniques are employed to meet specific research objectives, with each method offering unique advantages depending on the nature of the data and the goals of the study (Bhardwaj, 2019; Sarker and AL Muaalemi, 2022). For this study on prediction of FMD outbreaks in Uganda, the data sampling approach was strategically designed to enhance the performance of ML models used for predicting outbreaks. Given the dominance of FMD outbreaks in certain districts across Uganda, as illustrated in Figure 6, a purposive sampling strategy was adopted. Out of the total districts, 22 were carefully selected based on the frequency and intensity of outbreaks observed during the study period from 2011 to 2022. These districts, highlighted in Figure 7, were chosen because they had the highest recorded occurrences of FMD outbreaks, indicating that they are critical zones for the disease. By focusing on these high-frequency outbreak districts, the study aimed to ensure that the base ML model is trained on a robust and representative dataset. This approach not only provided the model with a substantial amount of relevant data but also provide a foundation for mitigating the risk of working with highly imbalanced datasets, which could undermine the model’s predictive performance. In districts with fewer outbreaks, data scarcity could lead to poor model training, resulting in less reliable predictions. Therefore, concentrating on districts with rich outbreak data was crucial for maintaining model accuracy. Moreover, these dominant districts are often referred to as “hotspots” in epidemiological research, as they are typically the sources of outbreaks that spread to neighboring regions. By prioritizing these hotspots in the sampling process, the study aimed to improve the generalizability of the predictive models. The insights gained from these key districts can be extrapolated to other areas, thereby enhancing the overall applicability of the model across Uganda. This targeted sampling strategy was fundamental in building a robust dataset that supports the development of high performing ML-based predictive model for FMD outbreak management.

The dataset for the 22 purposively selected districts comprised a total of 3,456 records, as detailed in Table 2. Within this integrated dataset, 96.15% of the records represented non-outbreak instances, while only 3.85% corresponded to FMD outbreaks. This distribution highlights a significant class imbalance, with a heavy skew towards non-outbreak records. Such an imbalance presents challenges for ML models, as they tend to become biased towards predicting the majority class in this case, non-outbreaks. This bias can lead to models that are less sensitive to detecting actual outbreaks, resulting in poorer predictive performance when it comes to identifying potential FMD outbreaks. Addressing this imbalance is therefore critical to enhancing the performance and reliability of the models.

The development and evaluation of the proposed CUP approach were conducted in two phases. Phase 1 (Section 3.1.2) involved designing and developing the algorithm. Phase 2 (Section 3.1.3) focused on evaluating the predictive performance of the CUP approach in mitigating distribution shifts for FMD outbreaks using the holdout validation dataset. These phases are detailed in the following sections:

#### 3.1.2 Phase 1: CUP development

In Phase 1, the study aimed to design and *develop the CUP approach to enhance the performance of the RF model under varying distributions for predicting FMD in Uganda*. This section discusses the various techniques adopted in designing and developing the approach. Table 3 shows the respective performances of the RF model in a stationary environment, the degradation under varying distributions, and the improved performance when subjected to the proposed CUP approach.

**Table 3:** Predictive performance improvement with the CUP approach.

	Test performance					Validation performance					CUP approach performance					Performance improvement Rate (%)				
Model	ACC	AUC	Recall	Precision	F1	ACC	AUC	Recall	Precision	F1	ACC	AUC	Recall	Precision	F1	ACC	AUC	Recall	Precision	F1
RF	0.92	0.97	0.94	0.90	0.92	0.46	0.58	0.03	0.24	0.06	0.99	0.84	0.74	0.86	0.79	114.13	45.17	2376.67	256.25	1218.33

ACC, accuracy; AUC, area under curve; CUP, calibrated uncertainty prediction.

##### 3.1.2.1 Experimental setup

The experiments for developing and evaluating the CUP approach for predicting FMD outbreaks were conducted using Python 3.11.4, leveraging its extensive ML libraries. The study was carried out within the Jupyter Notebook integrated development environment (IDE), utilizing a local ML platform optimized to use both GPU and CPU, significantly speeding up processing tasks. Key libraries such as Scikit-Learn, Pandas, NumPy, and Matplotlib were employed for data manipulation, model development, evaluation, and visualization, ensuring a streamlined and efficient workflow. This setup provided a solid foundation for accurate and reliable experimental results.

##### 3.1.2.2 Choosing RF as the baseline ML algorithm

The selection of RF as the baseline ML algorithm for performance improvement under varying distributions to predict FMD outbreaks in Uganda stemmed from the groundwork laid by our previous study (Kapalaga et al., 2024). The study explored seven ML models namely RF, Support Vector Machine (SVM), k-Nearest Neighbors (kNN), Gradient Boosting Machine (GBM), AdaBoost, Logistic Regression (LR), and Classification and Regression Tree (CART) to predict FMD outbreaks due to their diverse functionalities and strengths in handling various aspects of predictive modeling (Aghaei et al., 2021; Bansal et al., 2022; Cervantes et al., 2020; Choudhury et al., 2021; Joshi and Dhakal, 2021; Mienye and Sun, 2022; Touzani et al., 2018).

**Figure 8:**
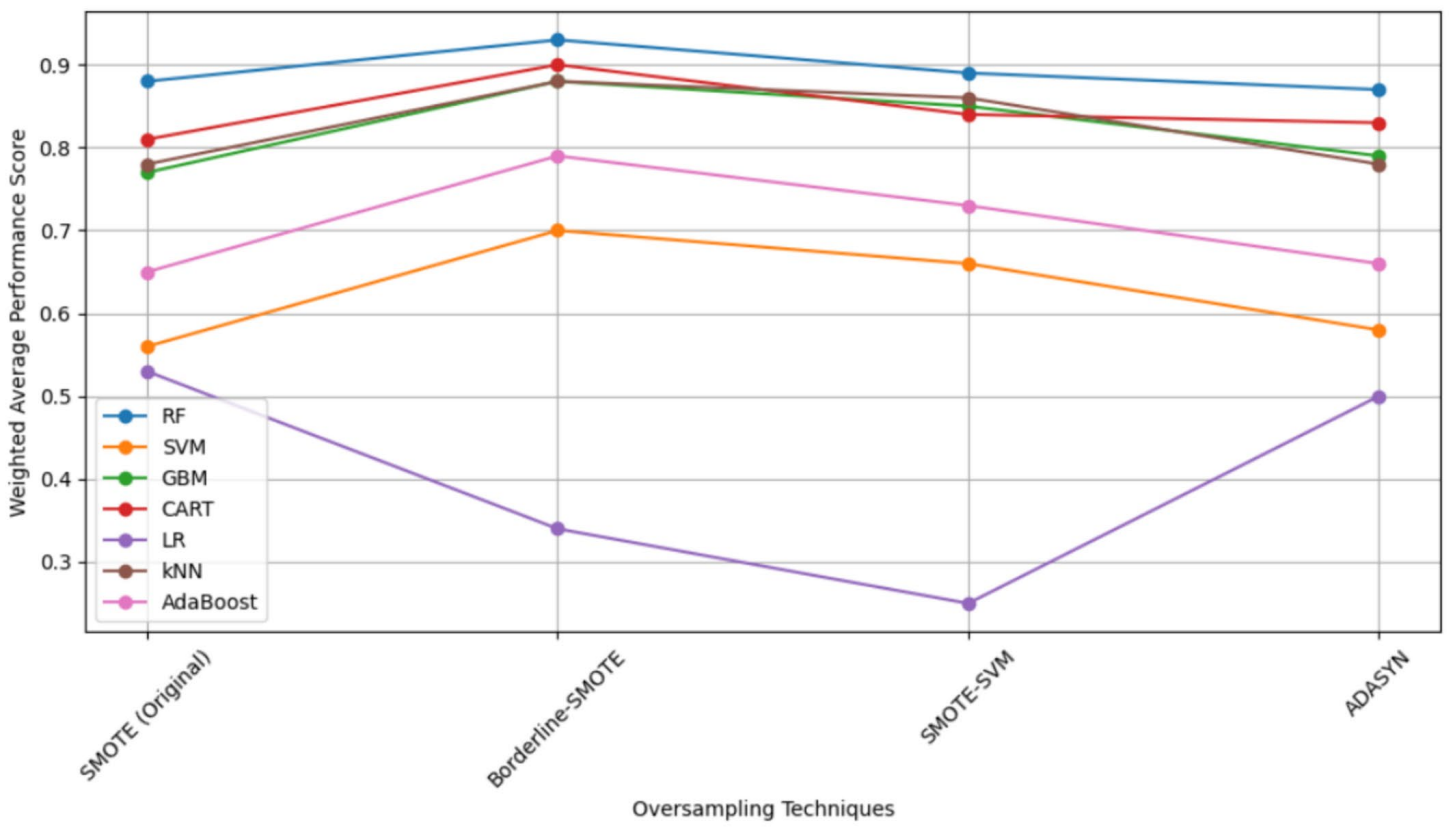
Model Performances across Oversampling Techniques with balanced dataset (Kapalaga et al., 2024). RF, random forest; SVM, support vector machine; kNN, k-nearest neighbors; GBM, gradient boosting machine; AdaBoost, adaptive boost; LR, logistic regression; CART, classification and regression tree; SMOTE, synthetic minority over-sampling technique; ADASYN, adaptive synthetic sampling.

In that study, RF was the best performing model under stationary environment as shown in Table 4 and Figure 8. The choice of RF is further supported by Punyapornwithaya et al. (2022) who explored the predictive capability of ML models in identifying FMD outbreaks in Thailand, through testing of various models, RF exhibited superior performance across all evaluation metrics. The superb predictive performance of RF is attributed to its ensemble nature where it integrates multiple decision trees to enhance performance (Choudhury et al., 2021). However, despite its superior predictive performance under stationary environment, RF demonstrated degradation in prediction of FMD outbreaks under varying distribution as depicted in Table 4 under validation performance, therefore this study aimed to enhance its predictive power by proposing the CUP approach which integrates techniques including borderline-SMOTE, active learning, probabilistic calibration and pseudo labeling.

**Table 4:** Weighted average performance scores of models for a balanced dataset (Kapalaga et al., 2024).

Weighted average performance scores				
Dataset before oversampling: no-outbreak—2.769; outbreak—111				
Balanced dataset after oversampling: no-outbreak—2.769; outbreak				
Model	SMOTE (Original)	Borderline-SMOTE	SMOTE-SVM	ADASYN
RF	**0.88**	**0.93**	**0.89**	**0.87**
SVM	0.56	0.70	0.66	0.58
GBM	0.77	0.88	0.85	0.79
CART	0.81	0.90	0.84	0.83
LR	0.53	0.34	0.25	0.50
kNN	0.78	0.88	0.86	0.78
AdaBoost	0.65	0.79	0.73	0.66

RF, random forest; SVM, support vector machine; kNN, k-nearest neighbors; GBM, gradient boosting machine; AdaBoost, adaptive boost; LR, logistic regression; CART, classification and regression tree; SMOTE, synthetic minority over-sampling technique; ADASYN, adaptive synthetic sampling. Bold values indicate the best-performing model across the various oversampling techniques.

##### 3.1.2.3 Integration of borderline-STOME, active learning, probabilistic calibration, and pseudo-labeling

The proposed CUP approach based on the data centric setting aimed to enhance RF’s performance by leveraging a combination of advanced techniques including borderline SMOTE, active learning, probabilistic calibration, and pseudo labeling tailored to handle imbalanced data, improve model calibration, and enhance generalization. In the following sections, the study delves into a detailed discussion on how these techniques were integrated to enhance RF’s performance in prediction of FMD outbreaks in the ever-evolving environment of Uganda.

###### 3.1.2.3.1 Mitigating class imbalance with borderline-SMOTE

**Figure 9:**
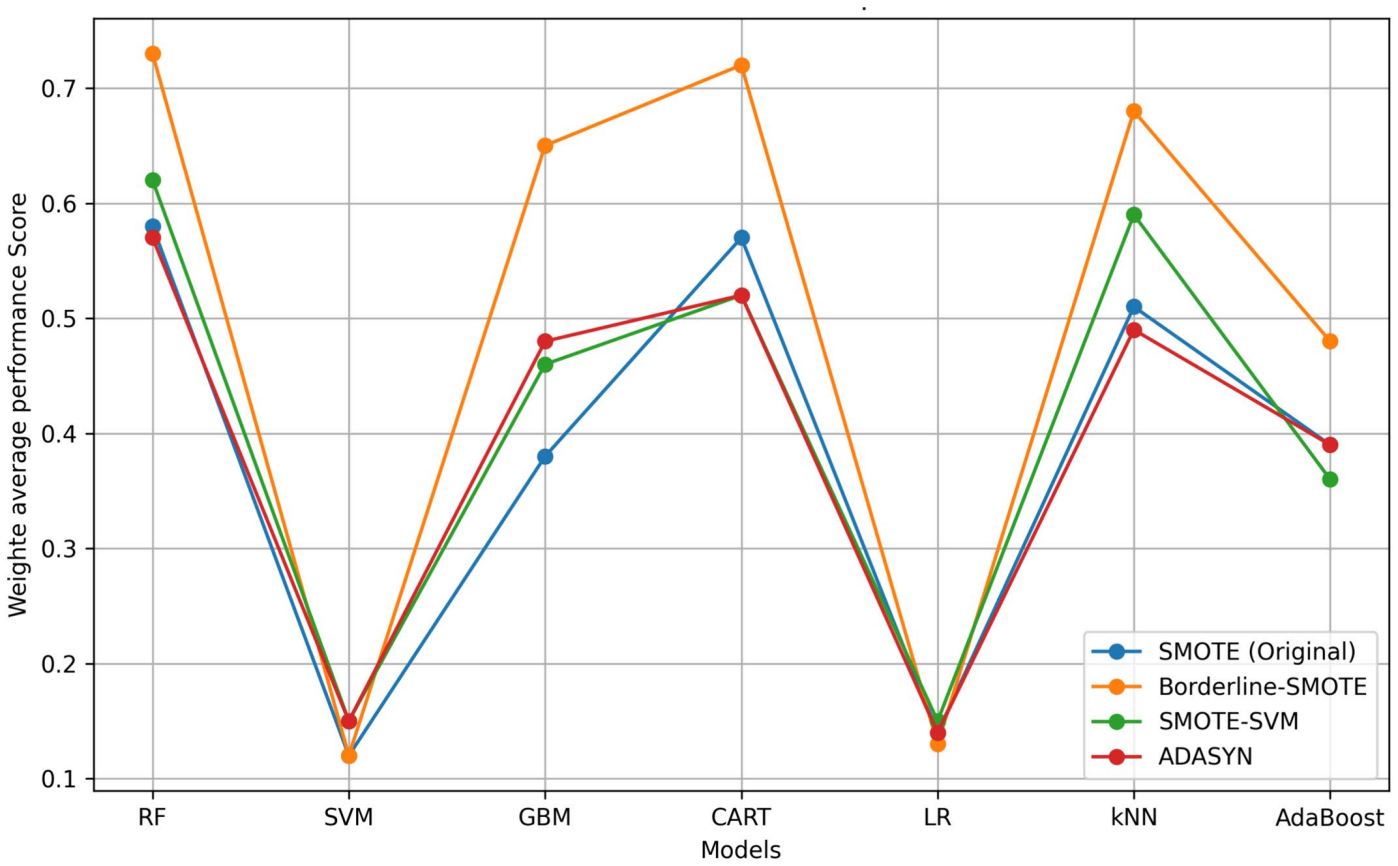
Comparative model performance across oversampling techniques. RF, random forest; SVM, support vector machine; kNN, k-nearest neighbors; GBM, gradient boosting machine; AdaBoost, adaptive boost; LR, logistic regression; CART, classification and regression tree; SMOTE, synthetic minority over-sampling technique; ADASYN, adaptive synthetic sampling.

The selection of the Borderline-SMOTE technique was informed by findings from our previous research (Kapalaga et al., 2024), where it was compared with three other data augmentation methods including original SMOTE, SMOTE-SVM, and ADASYN on the imbalanced FMD dataset. Two experimental approaches were taken: one involved oversampling the minority class (outbreaks) by a factor of 20 (Table 5 and Figure 9), and the other balanced the minority class to match the majority class size. As shown in Table 4 and illustrated in Figure 8, models trained on balanced datasets consistently outperformed those trained on imbalanced ones. Among the oversampling methods, Borderline-SMOTE emerged as the most effective (Figure 8). This success can be attributed to its focus on instances near the decision boundary between classes, where classification errors are most likely to occur. Unlike standard SMOTE, which generates synthetic samples across the entire feature space, Borderline-SMOTE specifically targets critical regions, thereby improving the model’s ability to accurately define the decision boundary. Given its strategic focus and proven effectiveness, Borderline-SMOTE was selected as the optimal technique for addressing class imbalance in our study.

**Table 5:** Weighted average performance scores of models with minority class oversampled by a factor of 20.

Weighted average performance scores				
Minority Class Oversampled by a Factor of 20				
Dataset before oversampling: no-outbreak—2.769; outbreak—111				
Dataset after oversampling: no-outbreak—2.769; outbreak—2.220				
Model	SMOTE (Original)	Borderline-SMOTE	SMOTE-SVM	ADASYN
RF	0.58	0.73	0.62	0.57
SVM	0.12	0.12	0.15	0.15
GBM	0.38	0.65	0.46	0.48
CART	0.57	0.72	0.52	0.52
LR	0.14	0.13	0.15	0.14
kNN	0.51	0.68	0.59	0.49
AdaBoost	0.39	0.48	0.36	0.39

RF, random forest; SVM, support vector machine; kNN, k-nearest neighbors; GBM, gradient boosting machine; AdaBoost, adaptive boost; LR, logistic regression; CART, classification and regression tree; SMOTE, synthetic minority over-sampling technique; ADASYN, adaptive synthetic sampling.

####### 3.1.2.3.1.1 Mathematical formulation of the borderline-SMOTE

To present the mathematical formulations of the Borderline-SMOTE technique in the context of predicting FMD outbreaks, it is essential to connect the general principles of Borderline-SMOTE with the specific variables and the RF model used for FMD prediction. The formulation involved three key steps:

- *Defining the problem context*: this step involved describing the task of predicting FMD outbreaks, the imbalanced nature of the dataset, and the necessity of addressing the minority class through resampling. In the context of Uganda, predicting FMD outbreaks requires using historical and environmental data. The main challenge lies in the dataset’s imbalance, where instances of FMD outbreaks are much fewer than non-outbreak instances, making it difficult for models to accurately predict outbreaks.
- *Formulating the mathematical model*: to achieve the formulation, there were three main steps involved as discussed below.

Step 1: Identifying the borderline FMD outbreak samples.

Let $X=\left\{x_{1},x_{2},\ldots ,x_{n}\right\}$ be the set of training samples, where each sample $x_{i}$ is a feature vector associated with either an FMD outbreak (minority class) or non-outbreak (majority class).

For each minority class sample $x_{i}$ (FMD outbreak):

- Find the k -nearest neighbors of $x_{i}$ in the training set, denoted as $N_{k}\left(x_{i}\right)$
- Let $k_{maj}$ be the number of majority class neighbors (non-outbreak) within $N_{k}\left(x_{i}\right)$ .

We define a sample $x_{i}$ as a borderline sample if:

$k_{maj}>\frac{k}{2}$ , meaning that the sample is surrounded by more non-outbreak cases than outbreak cases, placing it near the decision boundary.

Step 2: Generating synthetic samples.

For each borderline FMD outbreak sample $x_{i}$

- Randomly select a minority class neighbor $x_{\mathrm{neighbor}}$ from $N_{k}\left(x_{i}\right)$ .
- Generate a synthetic sample $x_{\mathrm{synthetic}}$ using linear interpolation:
$x_{\mathrm{synthetic}}=x_{i}+\lambda \cdot \left(x_{\mathrm{neighbor}}-x_{i}\right)$ , where $\lambda ∼\mathrm{Uniform}\left(0,1\right)$ is a random number between 0 and 1.

Step 3: Integrating with the FMD prediction model.

Let $X_{\mathrm{synthetic}}$ be the set of synthetic samples generated from borderline FMD outbreak cases.

The augmented training set $X^{\prime }$ used for training the FMD prediction model becomes:

$X'={XUX}_{\mathrm{synthetic}}$ , the new dataset X' is then used to train the predictive model to improve its ability to detect FMD outbreaks.

###### 3.1.2.3.2 Enhancing model confidence with active learning

Active learning is a subfield of ML that studies how an active learner model can best identify informative unlabeled instances and request their labels from some oracle, usually a human annotator (Settles, 2009). This study explored a pool-based active learning setting using the uncertainty sampling technique to query the uncertainty samples where the active learner is most uncertain about the instances. Using the predict_proba method, RF acted as the active learner trained with dataset from 2011 to 2018, generated probability predictions for target samples, which might *not accurately reflect the true likelihood of class membership*. The study aimed to augment the training dataset with challenging samples, thus improving the model’s robustness to varying distributions. Furthermore, the study computed the absolute scores by measuring the difference between these probabilities and 0.5 to quantify uncertainty. Utilizing the argsort method, the study sorted absolute differences to select instances with the highest uncertainty scores. Samples with absolute probability scores less than 3.5 were considered, indicating uncertainty around the 0.5 probability mark (Nguyen et al., 2022).

The study systematically evaluated the model’s uncertainty by scrutinizing the highest score for a given class on specific instances. The study selected instances with the lowest score among those in the active learning set, ensuring a thorough exploration of uncertainty. The integration of these strategies sought to equip the model with enhanced adaptability to varying data distributions, ultimately improving predictive performance and overall model robustness.

###### 3.1.2.3.3 Enhancing uncertainty estimates with probabilistic calibration

**Figure 10:**
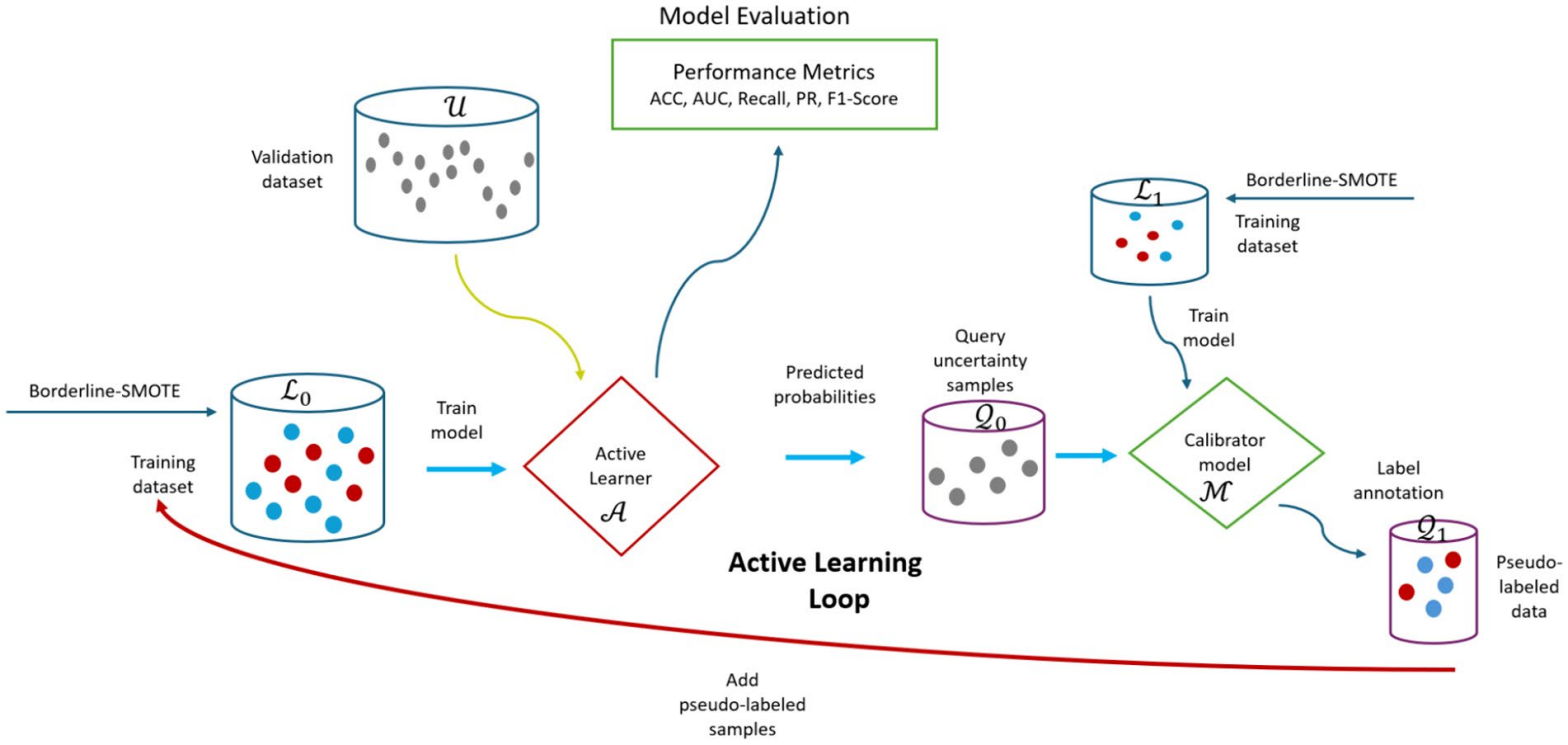
Visual overview of the CUP approach. ACC, accuracy; AUC, area under curve; PR, precision, $L_{0}$ , training dataset for training initial active learner ( A ); U , validation dataset; $Q_{0}$ , queried uncertainty samples; $L_{1}$ , dataset for training model calibrator ( M ); $Q_{1}$ , pseudo-labeled uncertainty samples.

To enhance prediction under distribution shifts, the study opted for probabilistic calibration technique to adjust the probabilities of the uncertainty samples to better align with the true probabilities. Probability calibration refers to refining the predicted probabilities generated by a ML model to improve their accuracy and reliability (Kuleshov et al., 2018). The study trained a logistic regression algorithm using dataset from 2019–2020 to act as the calibration layer for correcting the probabilities of uncertainty samples queried form the target unlabeled pool $Q_{0}$ (Figure 10). This study used the CalibratedClassifierCV class from scikit-learn for probability calibration. Specifically, Platt Scaling with the sigmoid method was employed. Platt Scaling is a logistic regression model trained to map the model’s raw scores output before applying the logistic function to calibrate probabilities (Bella et al., 2013). The study aimed to refine the predicted probabilities of challenging samples, aligning them more closely with their true probabilities.

###### 3.1.2.3.4 Pseudo-labeling with calibrated uncertainty probabilities

The process of pseudo-labeling involved assigning labels to the uncertainty samples based on their calibrated probabilities by the calibrator model using a thresholding method. The calibrated probabilities are compared to a chosen threshold value of 0.5 (default), where samples with probabilities above the threshold are assigned the label corresponding to the positive class (outbreak), and samples below the threshold are assigned the label corresponding to the negative class (non-outbreak). These pseudo labels are then utilized to retrain the active learner, with the goal of enhancing the overall predictive performance for FMD outbreaks under distribution shifts. This iterative approach aims to refine the model’s understanding of uncertainty and improve its ability to make accurate predictions under distribution shifts.

###### 3.1.2.3.5 The proposed CUP design

In the proposed CUP approach, the study employs a four-staged strategy as illustrated in Figure 10. The first stage is training the RF baseline model on source dataset (2011–2018) represented as $L_{0}$ to act as the active learner represented as A , and be utilized to predict the probabilities of the target unlabeled validation dataset (2021–2022) represented as U . The second stage is uncertainty selection, which involves using the predicted probabilities to select the most informative samples represented as $Q_{0}$ where active learner A is not confident. The third stage is the probabilistic calibration. At this stage, the queried uncertainty samples $Q_{0}$ are fed into the calibration model represented as M trained on dataset (2019–2020) represented as $L_{1}$ , adjusting their probabilities to better align with the true likelihood of outcomes using the sigmoid method. Still, at the same stage, the calibrated probabilities of the uncertain samples are converted into pseudo-labels. Finally, the fourth stage involves adding the pseudo-labeled samples represented as $Q_{1}$ to the initial training set $L_{0}$ for retraining the active learner. This repeats until the uncertainty samples are finished or once the model attains acceptable performance. We named this amalgamated approach as CUP and implemented as outlined in Algorithm 1.

The CUP algorithm (Algorithm 1) represents an iterative process of leveraging borderline-SMOTE, active learning, probabilistic calibration and pseudo-annotation to improve RF’s predictive performance in predicting FMD outbreaks in a non-stationary environment by utilizing uncertain instances in the validation dataset. The proposed algorithm utilizes the training dataset $L_{0}$ , calibration dataset $L_{1}$ to train the active learner A and model calibrator M respectively. The validation set is represented as U. In step 1, splits the training dataset $L_{0}$ into features and labels, and apply the borderline-SMOTE technique to enhance the representation of the minority class (outbreaks) by generating synthetic samples, outputting a balanced dataset $L\prime_{0}$ . In step 2, splits the calibration dataset, apply borderline-SMOTE outputting $L\prime_{1}$ . In step 3, $L\prime_{0}$ and $L\prime_{1}$ datasets are used in training the active learner A and model calibrator M respectively. Several iterations are performed represented as *T,* at every iteration in the CUP learning loop, the algorithm trains an active learn $A_{t}$ to predict on the features of augmented validation set (*X_target_*), the absolute difference is calculated to identify the most uncertain samples $X_{u}$ using the predicted probabilities. The trained model calibrator M predicts the labels for the uncertain samples, outputting $Q_{1}$ which is then added to the initial dataset $L_{0}$ to generate a new $L\prime_{0}$ dataset for training a new active learner $A_{t}$ and the cycle is repeated until desired results are achieved. Algorithm 1 indicates all the steps executed to achieve optimal performance for active learner (RF) in predicting FMD outbreaks in varying distributions.

####### ALGORITHM 1 CUP


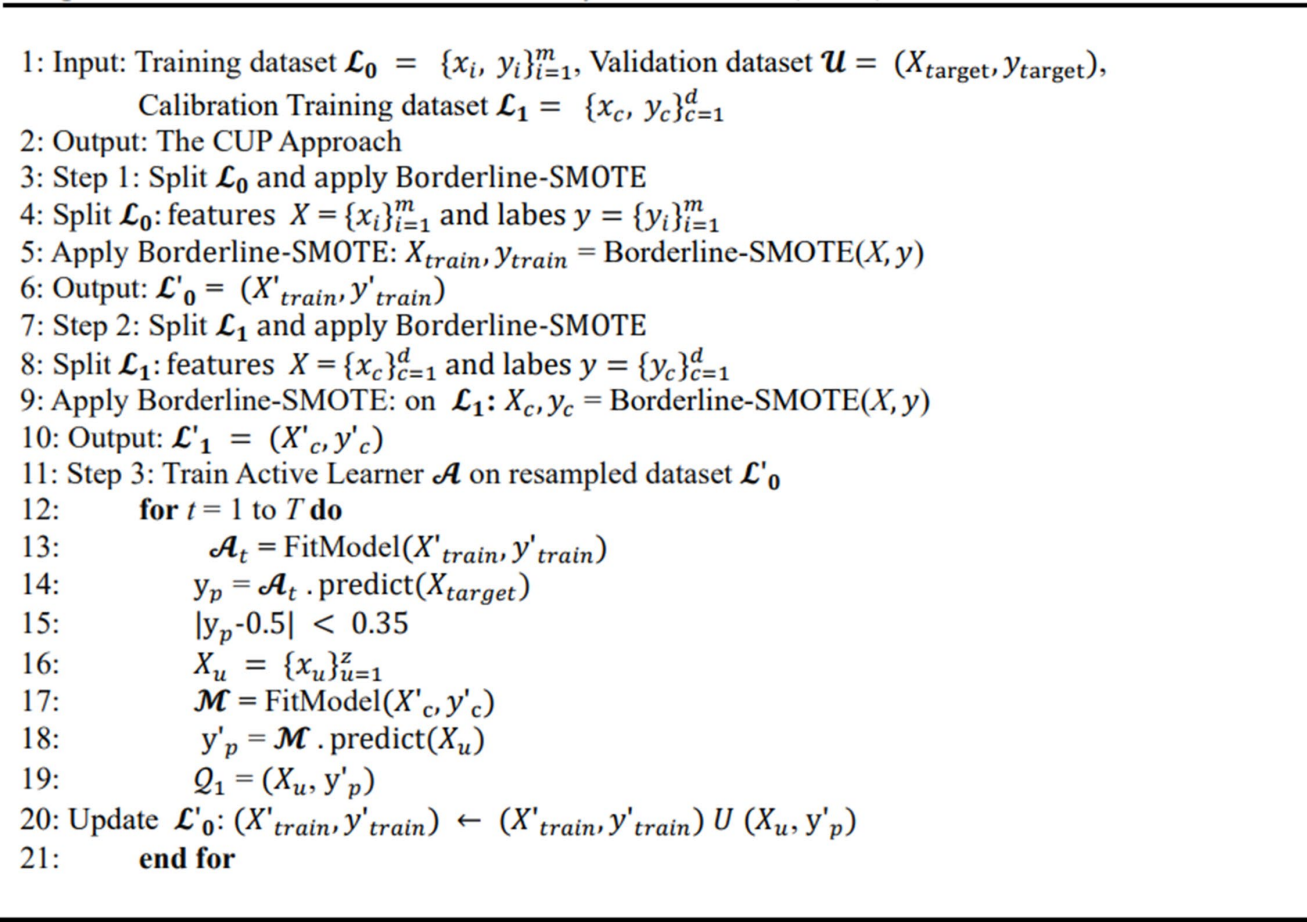


#### 3.1.3 Phase 2: evaluation

**Figure 11:**
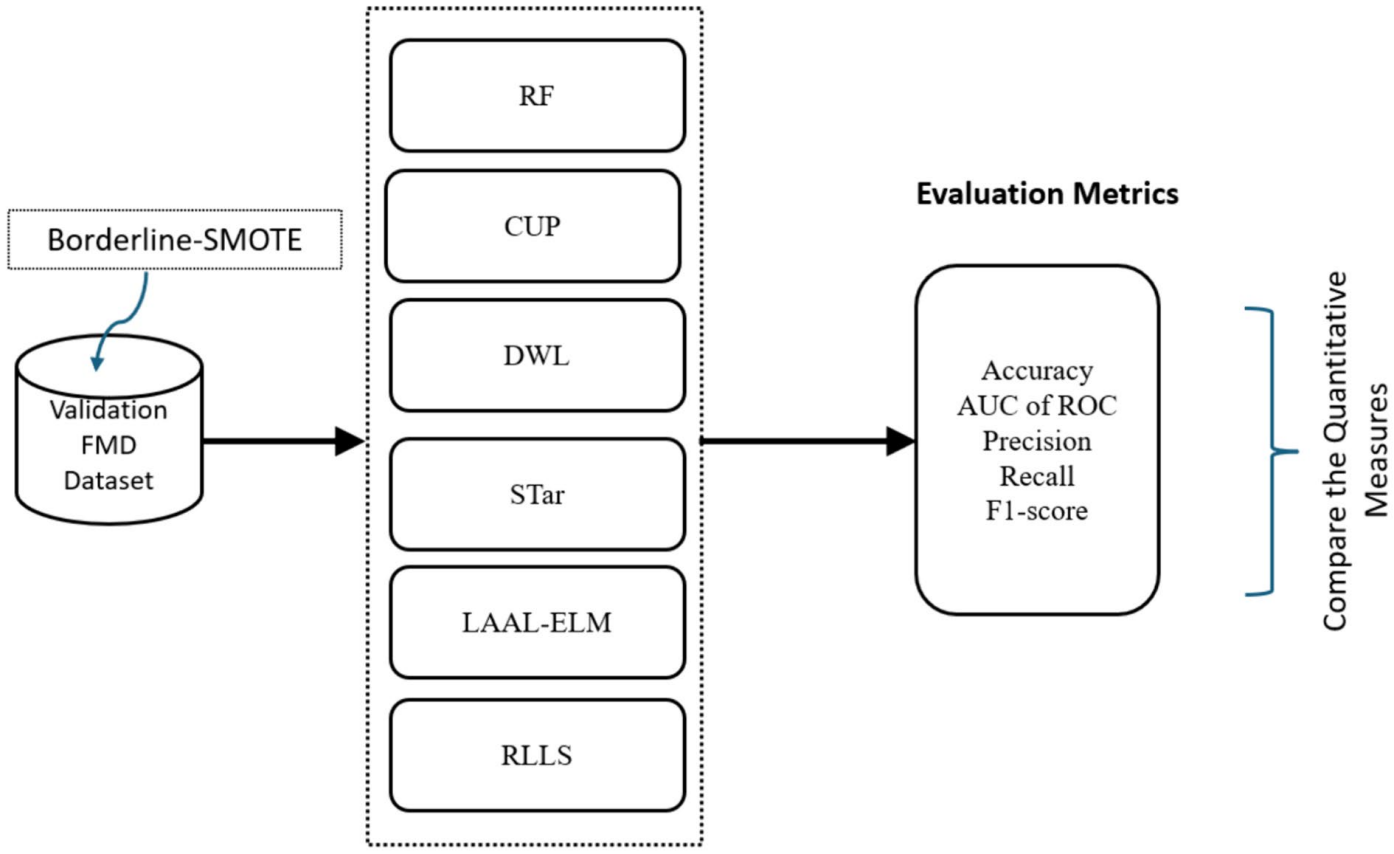
Workflow for evaluating performance of CUP with existing methods. AUC of ROC, area under curve of receiver operating characteristic; RF, random forest; DWL, dynamic weighted learning, STar, select TARgets; LAAL-ELM, less annotated active learning extreme learning machine; RLLS, regularized learning under label shifts; CUP, calibrated uncertainty prediction; FMD, foot-and-mouth disease.

In Phase 2, the study aimed to *evaluate the performance of the proposed CUP approach in enhancing the predictive power of RF in prediction of FMD outbreaks in Uganda under varying distributions*, the study utilized various classification performance metrics. These metrics included accuracy (ACC), AUC of ROC, precision (PR), recall, and F1-score which are discussed in detail under section 3.1.3.1. These metrics provided quantitative measures that allowed the study to compare the performances of CUP with five selected approaches known to mitigate distribution shifts (Figure 11) using the validation dataset (2021–2022). These approaches are RF (Balogun and Attoh-Okine, 2021), Dynamic Weighted Learning (DWL; Xiao and Zhang, 2021), Select TARgets (STar; Singh et al., 2021), Less Annotated Active Learning Extreme Learning Machine (LAAL-ELM; Yang et al., 2018) and Regularized Learning under Label shifts (RLLS; Azizzadenesheli et al., 2019).

##### 3.1.3.1 Performance evaluation metrics

To evaluate the performance of the proposed CUP approach in predicting FMD outbreaks under varying distributions, we utilized the following classification metrics: ACC, AUC, PR, Recall, and F1-score. These metrics were chosen to provide a comprehensive assessment of the model’s effectiveness in comparison to existing methods as discussed in sections 3.1.3.3 and 3.1.3.4. Additionally, these metrics were used to calculate the performance improvement rates of the CUP approach as discussed in Section 3.1.3.2.

##### 3.1.3.2 Performance improvement rates of CUP under varying distribution

To assess the performance improvement rates of the proposed CUP approach in predicting FMD under varying distributions, the study utilized the sequentially sampled target dataset (2021–2022). To quantify the performance improvement rates across all performance metrics, the study used the formula below.


$$\mathrm{Performance improvement rate}=\frac{\left(P_{\mathrm{target}}-P_{\mathrm{validation}}\right)}{P_{\mathrm{validation}}}\times 100\%$$

Where:

$P_{\mathrm{validation}}$ represents the performance for metric i , $P_{\mathrm{target}}$ represents the performance for metric i . For each performance metric, we calculated the difference between CUP’s performance (CUP approach performance) and the RF model’s performance under validation (Validation performance). This difference was then divided by the RF model’s performance under validation. The final result was expressed as a percentage (Table 3). This systematic approach allowed the study to evaluate improvement in performance metrics, serving as crucial indicator in assessing RF model performance improvement rates in prediction of FMD under varying distributions.

###### 3.1.3.2.1 Contribution of each component within the CUP approach

To determine the contribution of each component within the CUP approach, we conducted experiments by systematically removing individual components (borderline-SMOTE, active learning, probabilistic calibration, and pseudo-labeling) and evaluating their impact on overall performance. Each variant of CUP was assessed using the same FMD validation dataset and performance metrics detailed in Section 3.1.3.1.

The following CUP variants were tested:

- *CUP without Borderline-SMOTE:* to measure the impact of addressing class imbalance.
- *CUP without active learning:* to assess the role of active learning, especially in scenarios with limited labeled data.
- *CUP without probabilistic calibration:* to evaluate the importance of probabilistic calibration for prediction reliability.
- *CUP without pseudo-labeling:* to explore the contribution of pseudo-labeling in utilizing unlabeled data during training.

Comparing these variants against the complete CUP approach allowed us to quantify the significance of each component in achieving the observed performance improvements.

##### 3.1.3.3 Performance of existing methods using the FMD dataset

In this study, we evaluated the performance of five selected methods on the FMD dataset using a range of performance metrics as outlined in Section 3.1.3.1. This evaluation aimed to quantitatively assess how well these methods address class imbalance and distribution shifts in predicting FMD outbreaks in Uganda (Table 6). Specifically, 70% of the dataset from 2011 to 2018 was used as the training set, while the holdout dataset from 2021 to 2022 was used for validation.

**Table 6:** Performance of existing methods on FMD dataset.

Method	ACC	AUC	Recall	Precision	F1
RF	0.458	0.583	0.031	0.236	0.064
DWL	0.957	0.478	0.007	0.667	0.013
STar	0.823	0.467	0.136	0.035	0.056
LAAL-ELM	0.570	0.714	0.320	0.642	0.434
RLLS	0.358	0.382	0.097	0.064	0.001

ACC, accuracy; AUC, area under curve; RF, random forest; DWL, dynamic weighted learning; STar, select TARgets; LAAL-ELM, less annotated active learning extreme learning machine; RLLS, regularized learning under label shifts.

The selected methods for comparison represent diverse strategies for mitigating distribution shifts, a critical challenge in predicting FMD outbreaks. These methods were chosen based on their effectiveness in previous studies and their potential relevance to the FMD dataset:

- *RF:* a powerful ensemble technique known for its robustness in various ML tasks, including handling distribution shifts (Balogun and Attoh-Okine, 2021).
- *DWL:* this method dynamically adjusts model weights to better accommodate changes in data distribution, making it particularly effective in non-stationary environments (Xiao and Zhang, 2021).
- *Star:* star focuses on selective training, emphasizing critical samples that are most likely to improve model performance in the presence of distribution shifts (Singh et al., 2021).
- *LAAL-ELM:* this approach utilizes active learning with minimal annotated data, which is beneficial in scenarios where labeled data is scarce, and distribution shifts are prominent (Yang et al., 2018).
- *RLLS:* RLLS addresses label shifts through regularization techniques, providing a mechanism to handle changes in the distribution of output labels (Azizzadenesheli et al., 2019).

These methods were selected to ensure a comprehensive and balanced comparison with our proposed CUP approach, which integrates strategies including borderline-SMOTE, active learning, probabilistic calibration, and pseudo-labeling. By evaluating these established methods on the same FMD dataset, we provide a clear and direct comparison that highlights the strengths and limitations of each approach relative to the CUP method.

##### 3.1.3.4 Comparison of CUP approach performance with existing methods

The performance of the proposed CUP model was benchmarked against established methods known for handling distribution shifts as discussed under section 3.1.3.3 Performance of existing methods using the FMD dataset, offering a comparison to state-of-the-art strategies. The validation of CUP is essential to show that it either outperforms or is at least on par with these existing methods. This would solidify CUP’s position as a reliable and potentially more effective solution. The chosen methods have been tested across various domains and datasets, highlighting their generalization abilities. By comparing CUP against these established approaches, the study underscores its robustness and potential for application beyond FMD prediction. The comparative evaluation involved aggregating individual performance metric scores into a single metric, the weighted average performance score, which was then used to rank the models. The evaluation process followed these key steps.

- *Assign weights:* each performance metric was assigned an equal weight of 1, reflecting their equal importance in the study.
- *Calculate weighted scores:* each performance metric was multiplied by its assigned weight, and the resulting values were summed.
- *Compute weighted average scores:* the sum of the weighted scores was then divided by the total number of performance metrics to obtain the weighted average.

Therefore, the formula for calculating the weighted average score for n metrics is as follows:


$$\mathrm{Weighted average score}=\frac{\sum_{i=1}^{n}W_{i}\times M_{i}}{n}$$

Where:

$W_{i}$ represents the weight assigned to metric i ,

$M_{i}$ represents the value of metric i , and

n is the total number of metrics.

## 4 Results

In this section, the study reveals the research findings related to enhancing RF model predictive performance for FMD outbreaks in Uganda under varying distributions. The comprehensive investigation unfolds in two significant sections: assessment of predictive performance improvement rates with CUP approach under varying distributions, and evaluation of CUP performance in comparison with existing methods from the literature.

### 4.1 Predictive performance improvement with the CUP approach

**Figure 12:**
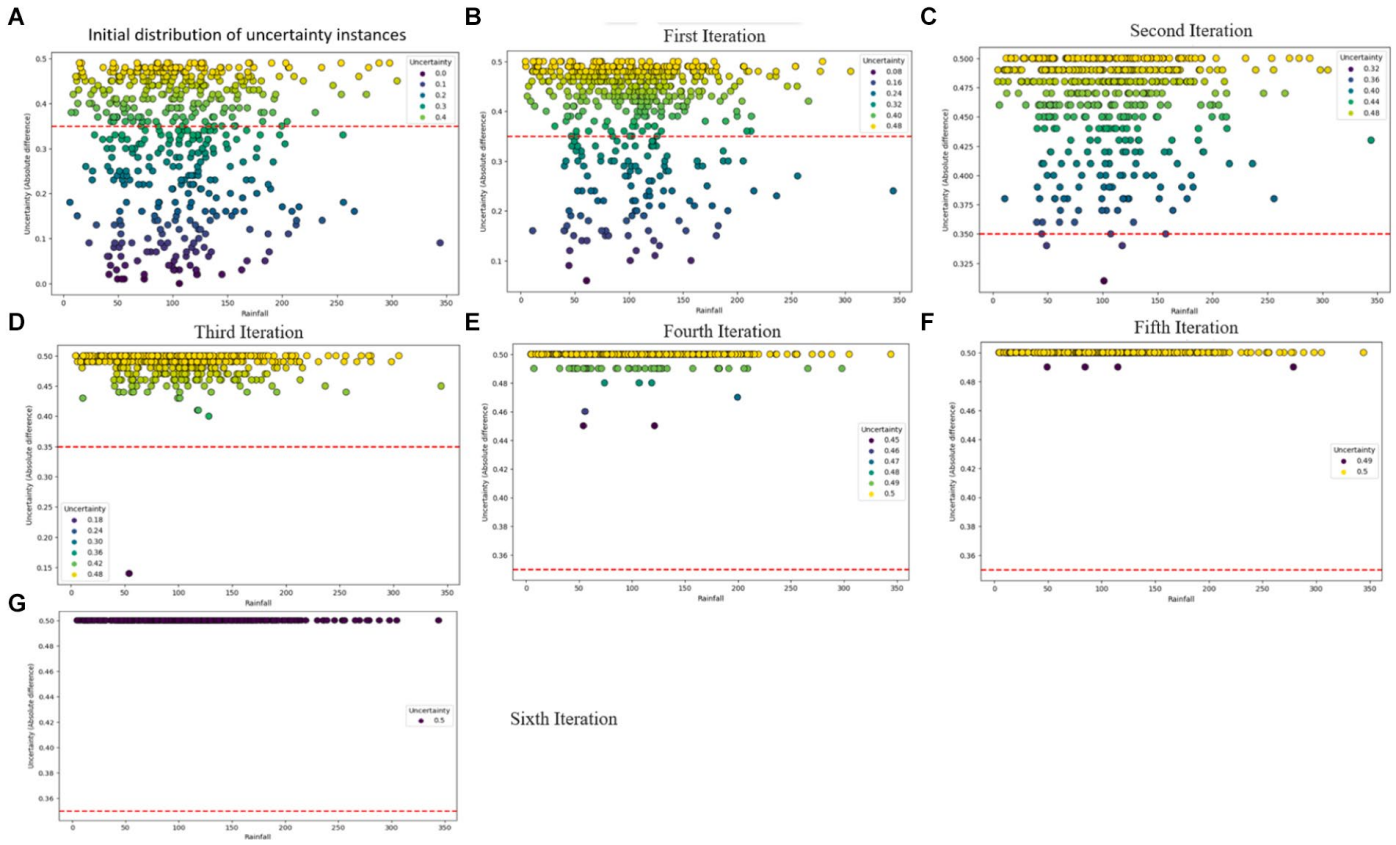
Visual overview of the iterative probabilistic calibration process applied to uncertainty instances. **(A)** Depicts the distribution of uncertainty samples before calibration, while **(B–F)** illustrate the status of uncertainty examples after iterative calibrations. **(G)** Showcases a scenario where uncertainty samples are perfectly calibrated.

The predictive performance of the CUP approach, as illustrated in Table 7 and Figures 12B–G showcases the impact of the proposed method through the iterative selection of the most uncertain instances for probabilistic calibration. Across the six iterations of active learning, the results demonstrate remarkable improvement, with probabilities approaching near-perfection. Figure 12A provides insight into the uncertain samples before calibration, displaying their absolute differences. By employing the absolute difference metric, the study focused on a pool-based active learning scenario, explicitly emphasizing the identification of uncertain instances for probabilistic calibration and subsequent fine-tuning.

**Table 7:** Performance of the CUP approach across six active learning iterations.

Iterations	ACC	AUC	Recall	Precision	F1-score
1	0.957	0.602	0.455	0.433	0.444
2	0.979	0.722	0.591	0.813	0.684
3	0.986	0.836	0.682	0.938	0.789
4	0.991	0.919	0.818	0.947	0.878
5	0.997	0.974	0.909	1.000	0.952
6	1.000	1.000	1.000	1.000	1.000
Average performance	0.985	0.842	0.743	0.855	0.791

ACC, accuracy; AUC, area under curve.

Table 3 illustrates the predictive performance of various RF model for FMD outbreaks across different evaluation levels. The model initially demonstrated excellent performance on the test dataset, as reflected in the “Test Performance” column. However, when the model was validated against dataset with varying distributions, the RF model exhibited a notable decline in performance, as shown in the “Validation performance” column of Table 3. Given the RF model’s limitations under varying data distributions, it was selected as the baseline model for comparison with the proposed CUP approach. The CUP approach aimed to address the challenges faced by the RF model, particularly under conditions of distributional shift.

**Figure 13:**
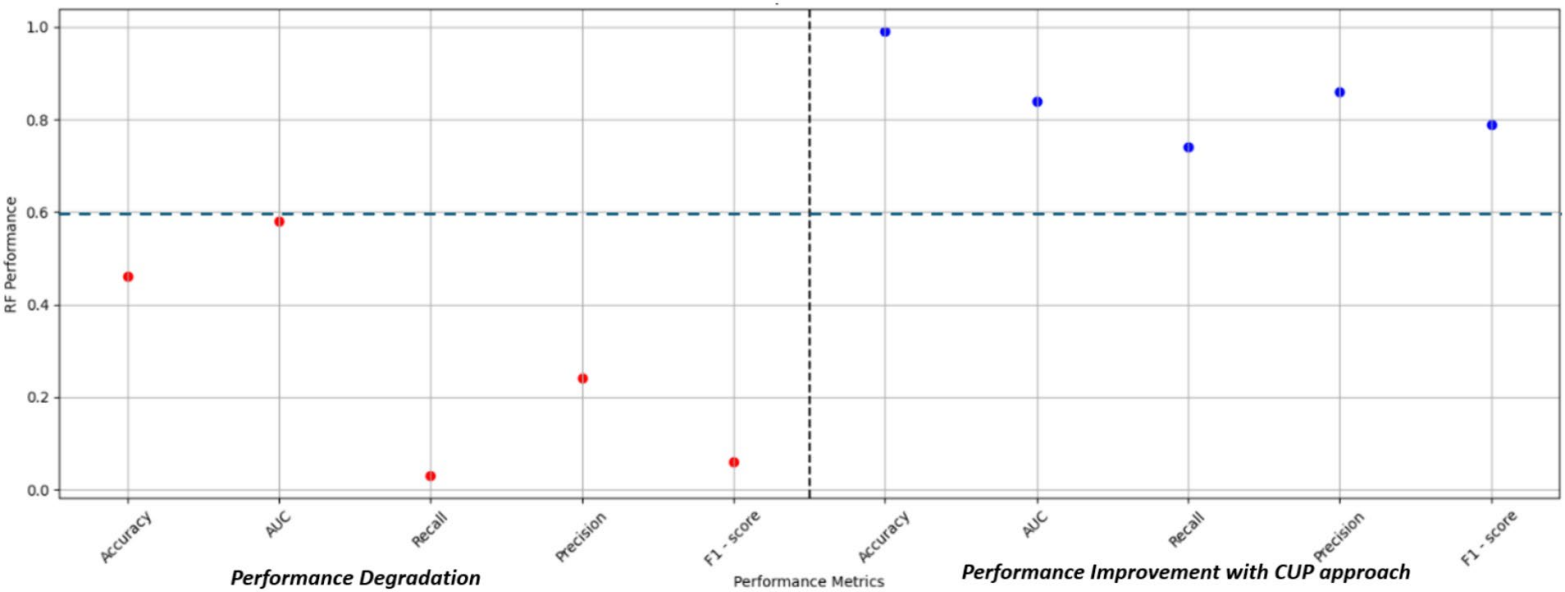
Performance improvement with the CUP approach. AUC, area under curve.

As detailed in Tables 7, 3, the CUP approach demonstrated substantial improvements in predictive performance metrics compared to the baseline RF model. Notably, key performance indicators such as Recall and F1-score showed significant percentage increases, indicating an enhanced ability of the model to correctly identify true positive instances of FMD outbreaks. These improvements in Recall highlight the CUP approach’s increased sensitivity, while the enhanced F1-score reflects a better balance between precision and recall, ultimately leading to more reliable predictions. Figure 13 further illustrates these performance gains, showcasing the effectiveness of the CUP approach in improving the RF model’s ability to predict FMD outbreaks. This is particularly crucial in the context of Uganda, where climatic conditions and other environmental factors are continually evolving, making accurate and reliable predictions of FMD outbreaks more challenging.

#### 4.1.1 Component-wise performance of the CUP approach

The performance of the CUP approach was assessed by systematically removing individual components and evaluating their impact on predictive performance. The removal of Borderline-SMOTE led to a dramatic decrease in the model’s ability to handle class imbalance, as evidenced by the extremely low precision, recall, and F1-score (7). Although accuracy remained high, it is misleading due to the model’s failure to effectively identify minority class instances. On the other hand, excluding active learning resulted in a reduction in overall accuracy compared to the full CUP approach. The model’s AUC was significantly higher, indicating better discriminatory power. However, precision, recall, and F1-score were notably lower, demonstrating the crucial role of active learning in enhancing the model’s performance in minority class prediction. Furthermore, the absence of probabilistic calibration led to a substantial drop in accuracy, indicating a significant loss in the model’s overall performance. Despite a relatively high AUC and precision, both recall and F1-score were lower, emphasizing the critical role of calibration in adjusting the model’s probability estimates to accurately reflect true class distributions.

The component-wise evaluation reveals that each element of the CUP approach plays a crucial role in enhancing model performance. Borderline-SMOTE is essential for managing class imbalance, as its absence severely impacts precision and recall. Active learning contributes to overall model accuracy and improves detection of uncertain instances, while probabilistic calibration is vital for enhancing probability estimation reliability and maintaining high accuracy. These results highlight the importance of each component in achieving the robust performance observed with the full CUP approach (Table 8).

**Table 8:** Component-wise performance of CUP approach.

	ACC	AUC	Precision	Recall	F1-score
Without Borderline-SMOTE	0.971	0.515	0.000	0.000	0.000
Without Active Learning	0.954	0.952	0.158	0.150	0.154
Without Probabilistic Calibration	0.654	0.797	0.752	0.460	0.571

ACC, accuracy; AUC, area under curve.

### 4.2 Evaluation of existing methods using the FMD dataset

In this section, we present the evaluation of five existing methods RF, DWL, STar, LAAL-ELM, and RLLS on the FMD dataset. The performance metrics used in this evaluation include ACC, AUC, Recall, Precision, and F1-score. These metrics provide a comprehensive understanding of each method’s ability to predict FMD outbreaks under varying distribution conditions. Table 6 presents the predictive performance across various metrics, which are further discussed below:

RF, known for its robustness in many classification tasks, exhibited limited effectiveness when applied to the FMD dataset. The model achieved an accuracy of 0.458 and an AUC of 0.583, suggesting moderate discriminative power. However, its Recall was notably low at 0.031, indicating a significant challenge in correctly identifying FMD outbreak instances. Precision stood at 0.236, while the F1-score was 0.064, reflecting the model’s struggle to balance recall and precision. These results suggest that while RF could identify some positive instances, its overall performance in handling the varying distributed FMD data was limited.

DWL, which dynamically adjusts model weights to account for distribution shifts, showed a high accuracy of 0.957. Despite this, its AUC was relatively low at 0.478, indicating limited capability in distinguishing between outbreak and non-outbreak instances. The model’s Recall was extremely low at 0.007, demonstrating a significant issue in detecting positive FMD cases. Interestingly, DWL achieved a Precision of 0.667, which is high but comes at the cost of an extremely low Recall. The resulting F1-score of 0.013 highlights the model’s poor balance between precision and recall, questioning its effectiveness in this specific application.

STar, designed for selective training, produced mixed results. It achieved an Accuracy of 0.823, which is relatively high, but its AUC was the lowest among the methods at 0.467. This suggests that while the model was able to classify the majority class effectively, it struggled with the minority class. The Recall was 0.136, indicating some ability to detect FMD outbreaks, though not strong enough for reliable predictions. Precision was particularly low at 0.035, leading to a modest F1-score of 0.056. These results imply that STar’s focus on selective training may not have been sufficient to handle the varying distributed nature of the FMD dataset effectively.

The LAAL-ELM method, which leverages less annotated data in an active learning framework, delivered a balanced performance across the metrics. It achieved an Accuracy of 0.570 and the highest AUC among the methods at 0.714, suggesting good overall discriminative ability. The Recall was 0.320, indicating a relatively better capacity to identify FMD outbreaks compared to other methods. Precision was also high at 0.642, and the F1-score was 0.434, the highest among the methods evaluated. These results indicate that LAAL-ELM effectively balanced recall and precision, making it the most reliable method for predicting FMD outbreaks in this dataset.

RLLS, which addresses label shifts through regularization, performed the weakest among the methods evaluated. It recorded an Accuracy of 0.358 and an AUC of 0.382, both of which are the lowest in this comparison. The model’s Recall was 0.097, suggesting poor sensitivity to FMD outbreak instances. Precision was also low at 0.064, resulting in an F1-score of just 0.001. These metrics highlight the challenges RLLS faced in adapting to the distribution shifts present in the FMD dataset, leading to an overall ineffective performance.

### 4.3 Comparative analysis of the CUP approach with existing methods

**Figure 14:**
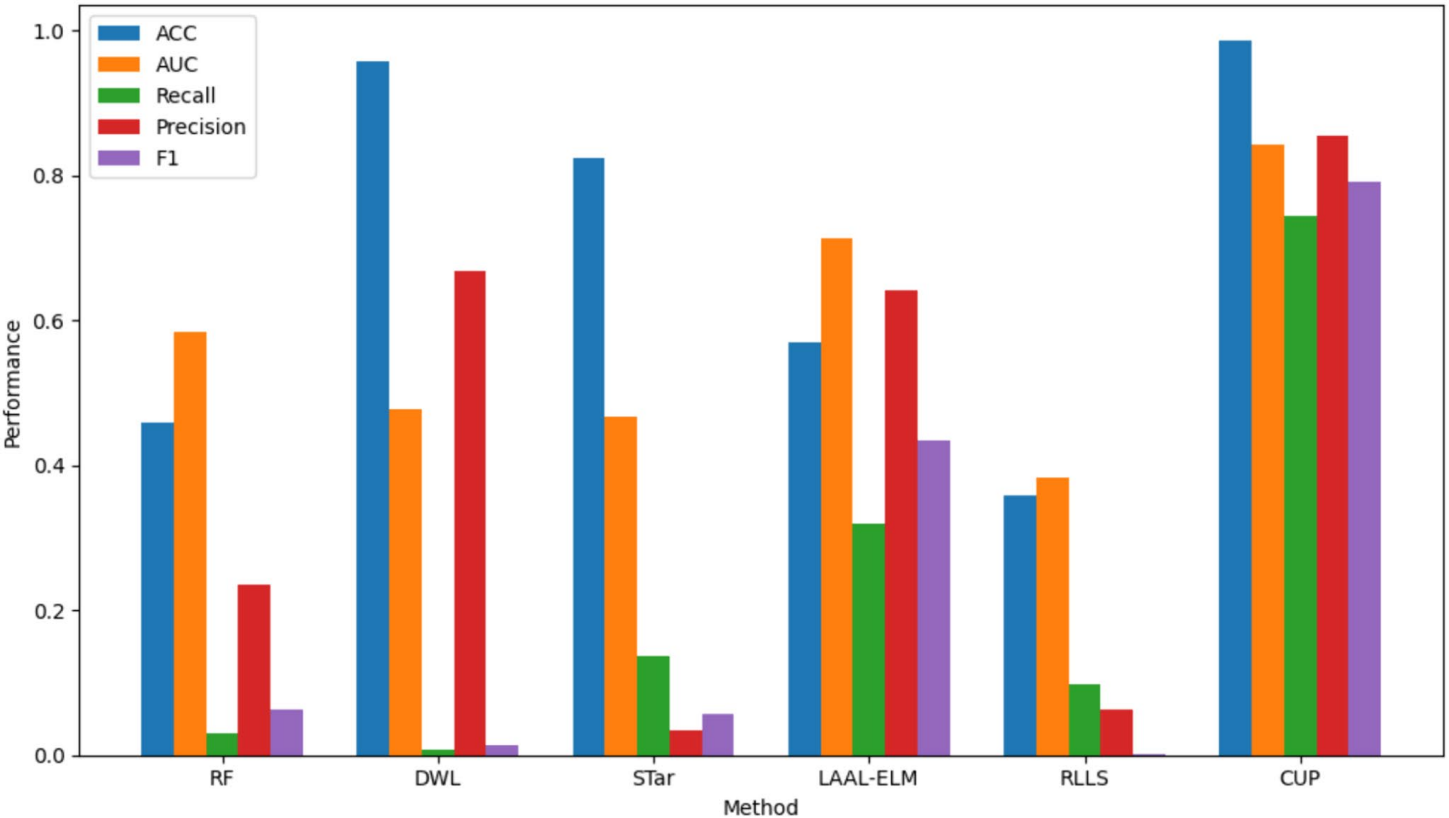
Comparative performance analysis of various methods. ACC, accuracy, AUC, area under curve, RF, random forest; DWL, dynamic weighted learning; STar, select TARgets; LAAL-ELM, less annotated active learning extreme learning machine; RLLS, regularized learning under label shifts; CUP, calibrated uncertainty prediction.

**Figure 15:**
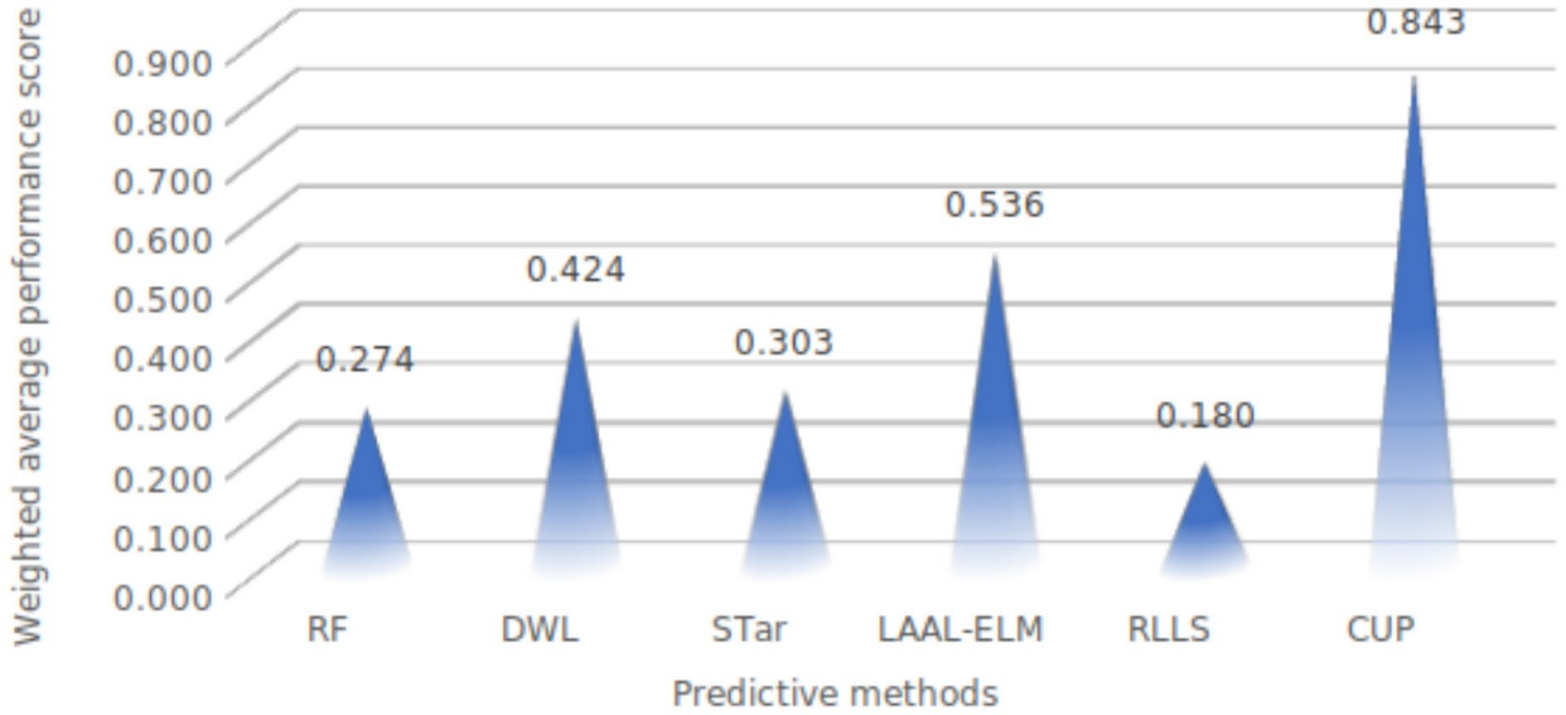
Weighted average performance of evaluated methods. RF, random forest; DWL, dynamic weighted learning; STar, select TARgets; LAAL-ELM, less annotated active learning extreme learning machine; RLLS, regularized learning under label shifts; CUP, calibrated uncertainty prediction.

In this section, we present a comparative analysis of the proposed CUP approach against the five selected stablished methods, including RF, DWL, STar, LAAL-ELM, and RLLS. The evaluation focuses on key performance metrics: ACC, AUC, Recall, Precision, and F1-score, with particular attention given to CUP’s ability to handle class imbalance and distribution shifts effectively (Table 9 and Figure 14). Furthermore, we calculated the weighted average performance score across all metrics for each method to identify the best-performing approaches (Figure 15).

**Table 9:** Comparative analysis of CUP performance with existing methods.

Method	ACC	AUC	Recall	Precision	F1
RF	0.458	0.583	0.031	0.236	0.064
DWL	0.957	0.478	0.007	0.667	0.013
STar	0.823	0.467	0.136	0.035	0.056
LAAL-ELM	0.570	0.714	0.320	0.642	0.434
RLLS	0.358	0.382	0.097	0.064	0.001
**CUP**	**0.985**	**0.842**	**0.743**	**0.855**	**0.791**

ACC, accuracy; AUC, area under curve; RF, random forest; DWL, dynamic weighted learning; STar, select TARgets; LAAL-ELM, less annotated active learning extreme learning machine; RLLS, regularized learning under label shifts; CUP, calibrated uncertainty prediction. Bold values indicate the best-performing model across the various performance metrics.

ACC: CUP achieved an impressive Accuracy of 0.985, significantly outperforming all other methods. The closest competitor, DWL, recorded an Accuracy of 0.957, but this came at the expense of extremely low recall and F1-score. The superior accuracy of CUP indicates its robust ability to correctly classify both outbreak and non-outbreak instances, making it highly reliable for predicting FMD outbreaks. In contrast, other methods like RF and LAAL-ELM, which achieved accuracies of 0.458 and 0.570 respectively, were less effective in distinguishing between the classes, particularly in the presence of imbalanced data.

AUC: CUP’s AUC of 0.842 further underscores its exceptional performance, indicating a strong ability to discriminate between FMD outbreak and non-outbreak cases across varying thresholds. This is notably higher than the AUCs achieved by the other methods, with LAAL-ELM being the closest at 0.714. The substantial gap in AUC highlights CUP’s superior handling of distribution shifts, ensuring that it maintains high discriminative power even under challenging conditions. Methods like STar and RLLS, which recorded AUCs of 0.467 and 0.382 respectively, struggled to perform well, particularly in cases where the minority class (FMD outbreaks) was severely underrepresented.

Recall: a critical metric for evaluating the performance of models in imbalanced datasets is Recall, which measures the model’s ability to correctly identify positive instances (FMD outbreaks). CUP excelled with a Recall of 0.743, indicating that it could identify a large proportion of actual outbreak cases. This is a dramatic improvement over the other methods, with LAAL-ELM being the next best at 0.320. The stark difference between CUP and methods like RF (0.031) and DWL (0.007) illustrates CUP’s effectiveness in overcoming the challenge of class imbalance, which often leads to under-prediction of minority class instances in traditional models.

Precision: CUP also demonstrated high Precision, scoring 0.855, which indicates its accuracy in predicting FMD outbreaks without a significant number of false positives. This precision was unmatched by other methods, with LAAL-ELM again being the closest at 0.642. The high precision, combined with its strong recall, suggests that CUP not only captures most of the true positive instances but also maintains a low false positive rate, making it an exceptionally reliable method for FMD outbreak prediction. In contrast, methods like STar, which recorded a Precision of 0.035, suffered from a significant number of false positives, reducing their overall effectiveness.

F1-Score: the F1-score provides a balanced measure of a model’s performance, taking into account both precision and recall. CUP achieved an outstanding F1-score of 0.791, far surpassing the other methods, with LAAL-ELM again trailing at 0.434. This high F1-score signifies that CUP effectively balances precision and recall, making it the most capable method for accurately and consistently predicting FMD outbreaks. The performance gap between CUP and methods like RLLS, which recorded an F1-score of just 0.001, highlights the substantial improvements CUP offers in handling both class imbalance and distribution shifts exhibited in FMD dataset.

#### 4.3.1 Weighted performance scores of methods on FMD dataset

The weighted average performance scores for the different methods reveal a clear distinction in effectiveness, with the CUP approach demonstrating exceptional performance as depicted in Table 9 . Among the methods evaluated, CUP achieved a remarkably high weighted average performance score of 0.843. This score significantly surpasses those of other techniques, highlighting CUP’s superior ability to handle the challenges of class imbalance and distribution shifts effectively. In comparison, the next highest score was recorded by LAAL-ELM, which attained a weighted average performance score of 0.536. While this score is notable, it is still considerably lower than CUP’s, indicating that LAAL-ELM, though effective, does not match CUP’s overall performance. Other methods such as DWL, STar, and RF had weighted average performance scores of 0.424, 0.303, and 0.274, respectively. These scores reflect their relative limitations in managing distribution shifts and class imbalance compared to CUP.

RLLS, with the lowest weighted average performance score of 0.180, demonstrates the least effectiveness among the evaluated methods, further underscoring CUP’s superior performance. The substantial gap between CUP and other methods underscores CUP’s robustness and its exceptional capability in achieving high accuracy, recall, precision, and overall balanced performance in predicting FMD outbreaks. Overall, CUP’s outstanding performance across all metrics positions it as the most effective method for addressing the complexities associated with class imbalance and distribution shifts in the FMD dataset, confirming its suitability as a leading approach in this domain.

## 5 Discussion of results

The main objective of this study was to *enhance predictive performance of RF model in predicting FMD outbreaks under varying distributions for enhanced preparedness in Uganda*, achieved through the proposed CUP approach that involved the integration of techniques including borderline-SMOTE, active learning, probabilistic calibration and pseudo-label annotation. Furthermore, the study evaluated the proposed CUP approach’s performance by utilizing five performance metrics including accuracy, AUC, recall, precision, and F1-score. The section presents a discussion of the study findings, contributions, limitations, and recommendations from this study.

### 5.1 Impact of component-wise contributions on CUP performance

The component-wise performance evaluation of the CUP approach provides valuable insights into the specific roles that each component plays in improving the model’s predictive capabilities. This evaluation highlights how the integration of each component including Borderline-SMOTE, active learning, probabilistic calibration, and pseudo-labeling collectively contributes to the overall success of the CUP approach.

The removal of Borderline-SMOTE resulted in a significant decline in the model’s ability to handle class imbalance, which was clearly reflected in the drastic reduction of precision, recall, and F1-score, despite the accuracy remaining relatively high. This discrepancy underscores the importance of considering more than just accuracy when evaluating models trained on imbalanced datasets. Borderline-SMOTE is particularly effective in addressing class imbalance by generating synthetic instances near the decision boundary where the model is most likely to make errors. By strategically focusing on these critical areas, the CUP approach, with Borderline-SMOTE, enhances the model’s ability to correctly classify minority class instances, which is essential for achieving a balanced and reliable predictive performance.

Active learning plays a pivotal role in the CUP approach by iteratively selecting the most uncertain samples from the validation set, which are then used to improve the model through calibration and pseudo-labeling. When active learning was excluded, there was a noticeable reduction in the model’s accuracy and predictive precision, particularly concerning minority class detection. While the AUC was slightly higher, indicating good discriminatory power, the reduction in precision, recall, and F1-score highlighted that active learning significantly contributes to the model’s ability to focus on challenging, uncertain samples, thereby enhancing its overall robustness. Active learning drives the model toward a more efficient learning process, ensuring that the most informative samples are used to refine the model iteratively, leading to improved performance on real-world, unseen data.

Probabilistic calibration is crucial for refining the model’s probability estimates, ensuring that the predicted probabilities align closely with the actual class distributions. The absence of probabilistic calibration resulted in a marked decrease in accuracy, despite maintaining relatively high AUC and precision. This drop in performance, particularly in recall and F1-score, highlights the importance of calibration in the CUP approach. Calibration ensures that the model’s predictions are not only accurate but also reliable, particularly when dealing with uncertainty in classification. By adjusting the probability estimates, probabilistic calibration reduces overconfidence in incorrect predictions and improves the model’s overall decision-making process. This leads to more balanced performance metrics and better generalization to new data.

Pseudo-labeling complements the active learning and calibration processes by providing additional training data, particularly from uncertain samples. These samples, once calibrated, are pseudo-annotated and added back to the training dataset. This iterative process helps the model to better understand the underlying data distribution and improves its ability to generalize. Without pseudo-labeling, the model misses out on valuable information that could have been leveraged to enhance its performance. The iterative retraining using pseudo-labeled samples helps to refine the model continually, improving its predictive power, especially in complex scenarios where labeled data is scarce or imbalanced.

#### 5.1.1 Superiority of CUP in mitigating distribution shifts in prediction of FMD outbreaks

To enhance the performance of the optimal baseline mode (RF) in predicting FMD outbreaks under distribution shifts, the study developed the CUP approach to address the challenge. To achieve a better performing CUP approach, the study integrated Borderline-SMOTE, active learning, probabilistic calibration, and pseudo-labeling techniques. This combination addressed class imbalance, queried uncertainty instances, calibrated their probabilities to align closer to the true values, and transformed the calibrated probabilities into pseudo-labels for retraining the baseline model. Subsequently, a CUP algorithm was developed to implement the approach in mitigating distribution shifts for predicting FMD outbreaks in Uganda.

The results highlight the exceptional performance of the CUP approach in mitigating distribution shifts and handling class imbalance for predicting FMD outbreaks. CUP achieved a remarkable Accuracy of 0.985, far surpassing the closest competitor, DWL, which scored 0.957. This indicates CUP’s superior ability to correctly classify both outbreak and non-outbreak instances. Additionally, CUP’s AUC of 0.842 demonstrates its strong capability in distinguishing between classes across various thresholds, outperforming other methods, including LAAL-ELM (0.714). The CUP approach also excelled in Recall with a score of 0.743, substantially higher than LAAL-ELM (0.320) and other methods, showcasing its effectiveness in identifying true outbreak cases. Its Precision of 0.855 further underscores its accuracy in making predictions while minimizing false positives, a notable improvement over LAAL-ELM and other methods. The F1-score of 0.791 achieved by CUP reflects a well-balanced performance between precision and recall, outshining the other methods, including RLLS, which had an F1-score of 0.001. The weighted average performance score of 0.843 for CUP, significantly higher than the next best score of 0.536 by LAAL-ELM, reinforces its superior overall performance. These results confirm CUP’s effectiveness and robustness in managing the challenges of class imbalance and distribution shifts, establishing it as the most reliable approach for predicting FMD outbreaks in the dataset.

### 5.2 Contributions of the study

The main objective of this study was to enhance the performance of RF in prediction of FMD outbreaks for enhanced preparedness in Uganda. The study’s contributions to methods and practice are as follows:

#### 5.2.1 Contribution of the study to methods

This study made significant contributions by devising a novel CUP approach based on the data-centric domain adaptation framework. This innovative methodology was rigorously evaluated, with the results demonstrating its notable superiority over conventional methods reported to tackle distribution shifts in ML domain. Through development and assessment, the study has enriched the methodological landscape, offering promising avenues for more effective and robust strategies in addressing distribution shifts within the ML domain.

#### 5.2.2 Contribution of the study to practice

By addressing the challenge of varying distributions in ML-based prediction of FMD outbreaks, this study significantly enhances preparedness for managing and controlling FMD in Uganda. The proposed CUP’s ability to provide timely and accurate predictions of potential FMD outbreaks under varying distributions offers valuable information to policymakers, farmers, and veterinary officers. This enables continuous surveillance of hotspots, early detection of outbreaks and facilitates optimal allocation of resources, ultimately improving the effectiveness of FMD management and control efforts in the country.

### 5.3 Limitations of the study

This section acknowledges the limitations encountered during the study and discusses their potential impact on the research findings:

- Computational resource requirements: The proposed CUP approach, which demonstrated superior performance in handling varying distributions, required significantly more computational resources. This increased demand for computational power may pose practical challenges in implementing the approach, particularly in settings with limited resources. Addressing this limitation by optimizing the computational efficiency of the CUP approach could enhance its feasibility and scalability for deployment in operational contexts. This may involve exploring techniques such as model pruning, and algorithmic optimizations to reduce processing time and resource utilization while maintaining predictive performance.
- Dataset-specific evaluation: Another limitation of the study is that the CUP approach was evaluated exclusively on the FMD dataset. This focus presents a gap in understanding its performance across different datasets. While CUP demonstrated impressive results in predicting FMD outbreaks, its effectiveness and generalizability to other domains remain untested. Future research should explore the application of CUP on various datasets to assess its robustness and adaptability in diverse contexts. This additional validation could provide a more comprehensive understanding of CUP’s capabilities and limitations beyond the FMD dataset.

## 6 Conclusion

The persistent challenge of FMD outbreaks poses significant threats to the livestock industry, communities, and economies, especially in developing countries like Uganda. Addressing this issue requires innovative approaches. Despite previous efforts to leverage ML for predicting FMD outbreaks, these studies often operated under stationary conditions, rendering the models vulnerable to varying distributions that significantly degrade their predictive performance. In this study, we proposed a CUP approach that integrates Borderline-SMOTE, active learning, probabilistic calibration, and pseudo-label annotation. Each component plays a distinct role: Borderline-SMOTE addresses class imbalance, active learning focuses on the most informative samples, probabilistic calibration ensures accurate probability estimates, and pseudo-labeling enhances the training dataset iteratively. The component-wise evaluation showed that removing any of these elements significantly degrades performance, emphasizing their collective importance in achieving the observed improvements. This approach aimed to enhance the performance of the RF model in predicting FMD outbreaks under varying distributions. Further evaluation demonstrated that the CUP approach significantly outperforms traditional methods, maintaining excellent predictive performance even when distribution shifts occur. The CUP approach’s iterative and integrated nature allows it to adapt and refine its predictions continually, leading to robust performance across various metrics. This innovative approach is crucial for managing FMD outbreaks in the endemic setting of Uganda. It facilitates continuous surveillance of potential outbreak hotspots, enabling early detection and optimal allocation of resources in resource-constrained regions of Uganda. The CUP method represents a significant advancement in predictive modeling for disease outbreaks, offering a more resilient and accurate tool for livestock management. Future work should focus on evaluating the performance of CUP across different datasets and conducting algorithmic optimizations to reduce processing time and resource utilization while maintaining predictive performance.

## Data Availability

The datasets presented in this study can be found in online repositories. The names of the repository/repositories and accession number(s) can be found in the article/Supplementary material.
